# Involvement of users and caregivers in mental healthcare decision-making within low-and middle-income countries: A scoping review

**DOI:** 10.1371/journal.pmen.0000712

**Published:** 2026-10-30

**Authors:** Action Amos, Sandra Jumbe, Thiyara Bandara, Sadiya Begum, Charlotte Hanlon, Sumeet Jain, Martyn Pickersgill, Eric Umar, Robert C. Stewart

**Affiliations:** 1 Division of Psychiatry, Institute for Neuroscience and Cardiovascular Research, University of Edinburgh, Edinburgh, United Kingdom; 2 Pan African Network of Persons with Psychosocial Disabilities, Kigali, Rwanda; 3 Wolfson Institute of Population Health, Queen Mary University of London, London, United Kingdom; 4 Department of Social & Health Sciences, Millenium University, Blantyre, Malawi; 5 Department of Psychiatry, School of Medicine, College of Health Sciences, Addis Ababa University, Addis Ababa, Ethiopia; 6 School of Social and Political Science, University of Edinburgh, Edinburgh, United Kingdom; 7 Kamuzu University of Health Sciences (KuHES), Blantyre, Malawi; 8 Centre for Biomedicine, Self and Society, Usher Institute, University of Edinburgh, Edinburgh, United Kingdom; University of Canterbury, NEW ZEALAND

## Abstract

Inclusive healthcare decision-making is a cornerstone of recovery-oriented mental health services, contributing to improved outcomes, enhanced autonomy, and reduced stigma. While substantial research on mental healthcare decision-making has been conducted in high-income countries, there is limited evidence from low- and middle-income countries (LMICs), where sociocultural norms, family structures, and health system constraints may shape decision-making differently. This review sought to synthesise evidence on service user and caregiver involvement in mental healthcare decision-making in LMICs and identify contextual factors influencing these processes. A scoping review was conducted following Arksey and O’Malley’s five-stage framework. English-language studies from 2008 to 2025 were identified through databases and grey literature searches. Eligible studies extracted examined the involvement of people with mental health conditions and/or caregivers in mental healthcare decision-making within LMICs. Two reviewers independently coded descriptions of involvement using an inductive content analysis approach. Three overarching themes emerged: (1) active user involvement, reflecting service users’ participation in and co-creation of decisions about their care; (2) family dominance, where family members significantly influenced or controlled decision-making processes; and (3) drivers of decision-making, including cultural norms, stigma, economic hardship, social relationships, and health system constraints. Overall, decision-making was predominantly shaped by family authority and contextual factors, with relatively limited evidence of shared or supported decision-making approaches. The search yielded 21,830 records, with an additional 46 studies identified through grey literature searches. Following screening, 36 studies were included. Studies were conducted across Africa (n = 12), Asia (n = 22), and the Middle East (n = 2). Mental healthcare decision-making in LMICs is embedded within complex family, cultural, and structural contexts that both enable and constrain service user participation. This review contributes to promoting a contextualised understanding of mental healthcare decision-making in LMICs.

## Introduction

### Background

Involving individuals with lived experience of mental health conditions in decisions about their care is fundamental to human rights, as enshrined in the United Nations Convention on the Rights of Persons with Disabilities (UNCRPD) [1] and within World Health Organisation (WHO) guidance on Meaningful Engagement of Persons with Mental and Neurological Disorders [2]. However, global implementation of this principle remains limited, particularly in low- and middle-income countries (LMICs), where colonial legacies and economic precarity continue to constrain meaningful involvement [3] [4].

The UNCRPD, the first legally binding instrument focusing on the rights of people with disabilities [1], requires that persons with disabilities enjoy legal capacity on an equal basis with others and that appropriate support be provided to exercise this right (Article 12). It promotes ‘inclusive’ decision-making by removing legislative, environmental, and attitudinal barriers [3], and is consistent with the social model of disability (which emphasizes that individuals are ‘disabled’ by societal structures rather than impairments) [5]. The UNCRPD aligns with the broader concept of participatory decision-making, ensuring affected persons are engaged in all processes that concern them [6].

Healthcare decision-making includes decisions about treatment choices, provider choices, timing of care, location of care, consent, support system choices, recovery and coping strategies made by individuals, families, clinicians, and traditional and faith healers [7]. In LMICs, communitarian values often shape decisions, prioritizing collective welfare and interconnectedness over individual preference [8–11]. Roles and responsibilities within families and communities strongly influence care trajectories, especially where formal systems are under-resourced. Despite growing recognition of frameworks like shared decision-making [12], substitute decision-making regimes that remove individuals’ legal capacity remain widespread and may contradict global commitments to person-centred and participatory care [3,13–17].

Decision-making in mental healthcare encompasses several conceptually distinct approaches that should not be presented as points along a single continuum. Shared decision-making generally refers to a collaborative process in which people using health services and healthcare professionals exchange information, consider available options and preferences, and participate jointly in decisions concerning care or treatment [14]. Supported decision-making, in contrast, is principally concerned with enabling individuals to exercise their legal capacity by providing the assistance they require to understand information, consider options, communicate their will and preferences, and make or implement decisions [1]. The United Nations Convention on the Rights of Persons with Disabilities (CRPD), particularly Article 12, establishes the right of persons with disabilities to recognition of legal capacity on an equal basis with others and calls for access to support in exercising that capacity [1]. Substitute decision-making occurs when another person makes a decision on behalf of an individual, including through formal arrangements such as guardianship or through informal practices in which family members, caregivers or professionals make decisions without the person’s own will and preferences determining the outcome [1,3].

These concepts are therefore analytically distinct, although they may intersect in practice. For the purposes of this scoping review, these concepts were operationalised separately. Where studies described the involvement of family members or caregivers but did not provide sufficient information to determine whether their role constituted support or substitution, this was coded as caregiver involvement rather than being assigned to either category.

Against this background, there is a need to better understand how different forms of decision-making are described and experienced in mental healthcare in LMICs. Rather than assuming that one approach predominates, this scoping review seeks to examine the extent to which shared, supported and substitute decision-making approaches are reported, the actors involved in decision-making, and the contexts and factors that shape their involvement. This includes attention to the roles of persons with mental health conditions, caregivers, healthcare professionals and other family or community actors

Caregiver involvement also warrants particular attention because decision-making in mental healthcare may extend beyond interactions between the individual and healthcare professionals. Family members and other informal caregivers may play important roles in care-seeking, treatment decisions, consent, medication management, referral, admission and responses to periods of acute distress. WHO guidance recognises that decision-making may occur informally within family environments and that family members may provide different forms of support to people using mental health services [2,3]. This is particularly relevant in settings where mental healthcare is embedded within family and community relationships and where access to formal mental health services may be limited. In this review, the term **caregiver** primarily refers to informal caregivers, including family members and other persons who provide ongoing care unpaid, support or assistance to a person with a mental health condition. Professional healthcare providers are considered separately as healthcare actors unless a study explicitly identifies them as caregivers.

## Methods

This scoping review was conducted using processes established by Munn [18] and revised by Bougioukas [19]. A rigorous approach to literature searching and analysis was guided by Arksey and O’Malley’s [20] five-stage methodological framework revised by Levac [21]. This involves: (i) identifying the research question, (ii) identifying relevant studies, (iii) selecting eligible studies, (iv) charting the data, and (v) collating and summarizing the results. We conducted the review in compliance with the PRISMA Extension for Scoping Reviews (PRISMA-ScR) guidelines [22]. The protocol was registered with the Open Science Framework (OSF) https://doi.org/10.17605/OSF.IO/WM2UX. The scoping review team was composed of experts with diverse and complementary skills, ensuring a rigorous and comprehensive review process. The team has geographical expertise of working in Asia and Africa [23]. A university librarian advised on the development of a comprehensive search strategy to capture key articles. Content experts (AA, with SJain, CH, MP, RCS, LMT) determined the selection of search terms and keywords, developed the data extraction form, refined the analytical approach, and interpreted the findings. Methodological expert (SJumbe) ensured ongoing alignment with the research questions and research practice.

### Defining the research question

This scoping review aimed to map existing literature on how service users and caregivers have been involved in mental healthcare decision-making processes across home and healthcare (formal and informal) settings. Specifically, it sought to answer the research question:’How have persons identified as having mental disorder and caregivers in low- and middle-income countries (LMICs) been involved in mental healthcare decision-making processes across home and healthcare (formal, and informal) settings?’ The review also aimed to identify contextual influences on meaningful engagement [2], highlight research gaps, and offer future research recommendations. The research question was refined using the PCC (Population, Concept, Context) framework recommended by the Joanna Briggs Institute [24], where: the Population included persons identified as having mental health conditions and their caregivers in LMICs; the Concept focused on their involvement in mental healthcare decision-making; and the Context encompassed formal and informal healthcare settings (including homes, traditional or faith healing sites) (See Table 1 below). For this scoping review, the term’involvement’ is used to encompass patient-centred care, engagement, involvement, and partnership, aligning with broader literature [25,26]. Additionally, the term’service users’ is used interchangeably with ‘persons identified as having mental health conditions’.

**Table 1:** Inclusion and exclusion criteria.

	Inclusion Criteria	Exclusion Criteria
Concept/Focus	Studies or reports on user and caregiver engagement, involvement, and participation in mental healthcare decision-making within the home, and formal and informal healthcare settings	Studies not focusing on mental health.
Population	Persons diagnosed with mental health conditions and caregivers	Individuals who are not identified persons with a mental health disorder and caregivers who are not providing support to someone with a mental health condition
Context	Low- and middle-income countries (World Bank categorization at the time of publication of the study).	High-Income Countries
Language	Studies published in English	Studies published in other languages
Year	Year limit from 2008 to 2025	Publication date outside the specified time frame.

### Identification of relevant studies

Initial searches were conducted in the Ovid Medline, Embase, PsycINFO, Social Science Citation Index, Global Health, and SCOPUS databases. The search was limited to English-language articles published between 2008 and 2025, aligning with the entry into force of the UN Convention on the Rights of Persons with Disabilities (UNCRPD) in 2008. The lead reviewer (AA) developed the search strategy in consultation with a specialist librarian and the wider review team (see S1 Appendix).

Records were screened against the inclusion and exclusion criteria outlined in Table 1, and duplicate records were removed. Following this process, 10,986 studies were identified for further screening. In the second stage of screening, the abstracts of the remaining studies were reviewed collectively by three reviewers to assess relevance and inclusion based on the predefined eligibility criteria. For studies where consensus was not achieved, SJumbe acted as an adjudicator and made the final inclusion decision.

Although the review primarily focused on studies involving persons with mental health conditions and caregivers, studies involving healthcare professionals were also eligible where they provided direct insight into how user and caregiver involvement in decision-making was facilitated, constrained, negotiated, or experienced within mental healthcare settings. Although this scoping review did not seek to test theory, the synthesis was informed by the concept of relational autonomy as articulated by Pickersgill [27]. Relational autonomy recognises that individuals exercise agency within social, institutional, and interpersonal relationships rather than in isolation. This perspective was used as an interpretive lens during data synthesis to examine how studies described the factors that enabled or constrained the involvement of persons with mental health disorders in decision-making across different contexts.These studies were included because in healthcare more generally [2,27,28] decision-making is inherently relational and often occurs within interactions between service users, families, and providers.

### Data extraction and analysis

Data extraction was conducted independently by two reviewers (AA and SJumbe) using a standardized Excel data extraction sheet developed for this scoping review. Extracted information included study characteristics, setting, participants, and descriptions of decision-making processes involving persons with mental disorders. Extracted data were systematically coded and categorized using content analysis [29], alongside an interpretative examination of how reported practices reflected underlying decision-making dynamics, including power relations and exclusion. This approach was particularly suited to the heterogeneous nature of the literature and the absence of standardized terminology across studies. Explicit references to ‘decision-making involvement’ were absent in many included studies. Instead, decision-making processes were often implied through descriptions of actions, roles, interactions, and care pathways rather than directly articulated. Shared, supported and substitute decision-making were treated as distinct concepts [1,3,14]. Where caregiver or family involvement was reported without sufficient information to determine whether the role was supportive, collaborative or substitutive, the study was coded as reporting caregiver involvement without assigning it to a specific decision-making category [1,2,14]

Given the diversity of study designs, participant groups, and mental health conditions represented in the included literature, the purpose of synthesis was not to compare outcomes across diagnoses or generate pooled estimates. Instead, analysis focused on identifying recurring patterns relating to involvement in decision-making, irrespective of diagnosis, setting, or methodological approach. This approach is consistent with the objectives of scoping reviews, which seek to map the breadth and nature of available evidence rather than determine effectiveness or causality.

## Results

### Study selection

A systematic study selection process was undertaken to identify literature relevant to the review objectives. A total of 21,830 records were identified through database searches, with an additional 46 records identified through other sources. Following the removal of 10,986 duplicate records, 10,890 records were available for title and abstract screening. Records were screened against the predefined inclusion and exclusion criteria, with 10,749 records excluded at this stage because they did not include participants with mental disorders and/or were not conducted in low- and middle-income countries (LMICs).

The full texts of 141 articles were subsequently assessed for eligibility. Of these, 105 articles were excluded because they did not explore decision-making, resulting in 36 studies meeting the eligibility criteria and being included in the scoping review. The overall study selection process is presented in Fig 1.

**Fig 1:**
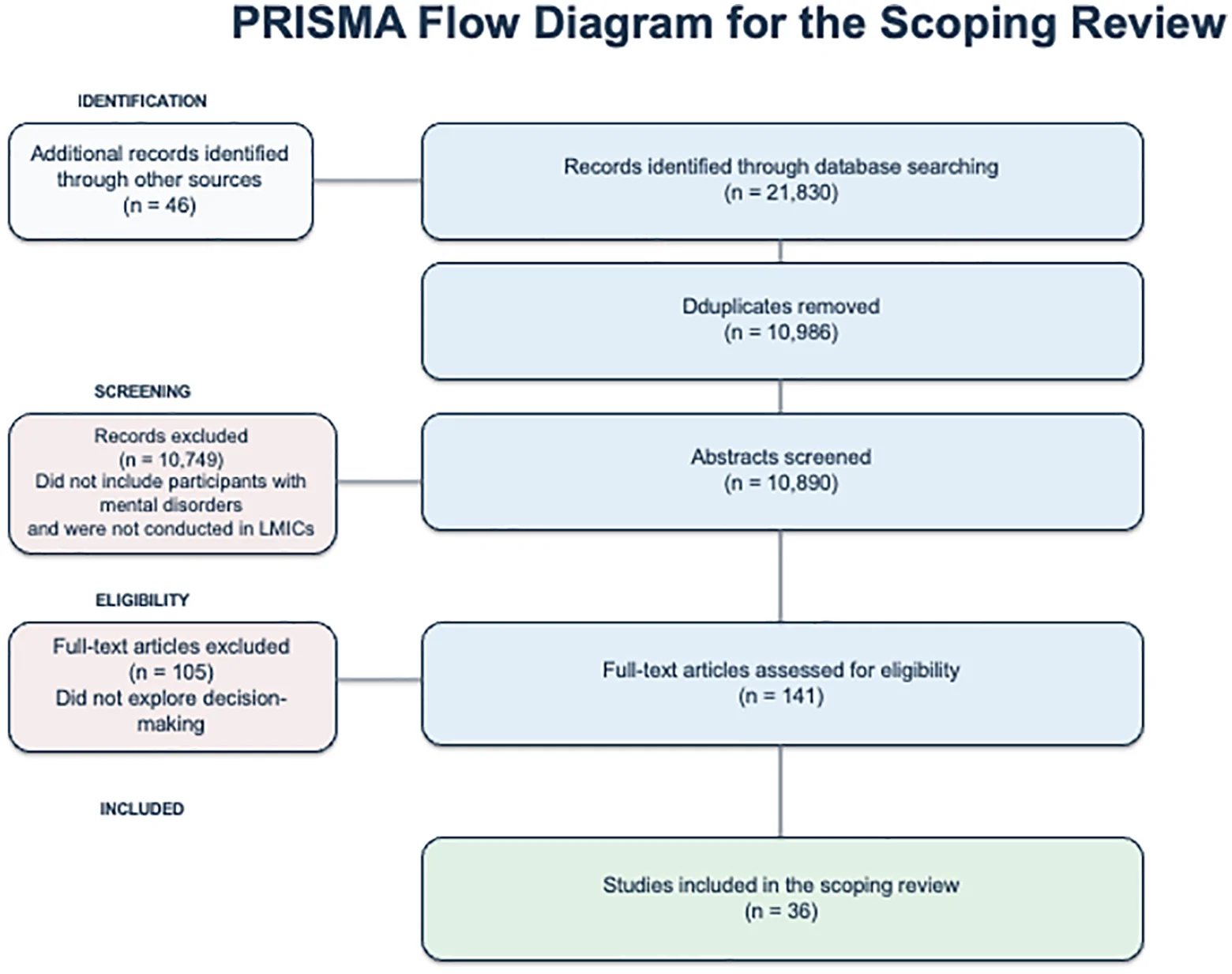
The PRISMA flow diagram showing the review process and results.

### Study characteristics

The included studies were conducted in Africa (n = 12), Asia (n = 22), and the Middle East (n = 2).

Most studies were conducted in Asia, primarily in South and Southeast Asia, including India, Nepal, Sri Lanka, Cambodia, Indonesia, Malaysia, Pakistan, China and Iran. These commonly examined pathways to care, family involvement in decision-making, and explanatory models of mental illness. They paid particular attention to the influence of cultural beliefs, stigma, and access to services on treatment decisions.

In Africa, studies were conducted in countries that primarily included Ghana, Nigeria, Malawi, South Africa, Zambia, Botswana, Ethiopia, and the Gambia. Studies from African contexts predominantly explored family dominance in decision-making, caregiver burden, stigma, and system- and governance-level challenges affecting mental health service delivery. In these, families frequently emerged as primary decision-makers, particularly in contexts of limited-service availability and weak institutional support.

Further studies were conducted in the Middle East; specifically, Lebanon. These focused on power dynamics, consent processes, and service-user participation within psychiatric services. They offered insights into how legal frameworks, professional authority, and family involvement intersect to shape mental health decision-making.

Collectively, the included studies employed a range of research designs. Qualitative designs were the most common (n = 19), which included phenomenological, descriptive qualitative, ethnographic, and interview-based approaches. These methods were primarily used to explore lived experiences, family roles, power relations, and implicit decision-making practices, particularly in contexts where decision-making involvement was not explicitly articulated.

Cross-sectional quantitative designs were used in 16 studies. These typically employed structured questionnaires or surveys to examine pathways to care, shared decision-making practices, caregiver involvement, and access to services. One study adopted a mixed-methods design, integrating quantitative and qualitative approaches to capture both experiential and structural dimensions of mental health decision-making.

As indicated, the geographical distribution of studies was uneven, with a concentration in countries from Asia and Africa. This pattern likely reflects differences in research capacity, availability of mental health research funding, publication practices, and the consolidation of mental health system strengthening initiatives, rather than the actual distribution of involvement practices across LMICs. Consequently, caution should be exercised when generalising findings to countries or regions that remain underrepresented in the literature.

Study settings reflected both formal health systems and informal or community-based care pathways. Most (n = 23) studies were conducted in healthcare facilities, including hospitals, psychiatric units, outpatient clinics, health centres, and tertiary care facilities. These studies often examined clinician-led decision-making, consent practices, and service-user participation within biomedical systems.

Some (n = 10) studies were conducted in community or home-based settings, highlighting family-led care, informal decision-making, and non-institutional pathways to care. A small number of studies (n = 3) were conducted in alternative or non-clinical settings, such as faith-based prayer camps or teaching institutes. These illustrated the influence of religious, educational, and cultural institutions on mental health decisions. Across the included literature, study settings frequently overlapped, with decision-making processes occurring across homes, communities, and health facilities, rather than within a single bounded setting.

Of the 36 included studies, n = 11 were published between 2008 and 2018, n = 18 between 2019 and 2023, and n = 7 between 2024 and 2025. This indicates a marked increase in scholarly attention to mental health decision-making, participation, and family involvement in LMIC contexts in recent years (see Table 2 below).

**Table 2:** Characteristics of included studies on decision-making involvement in mental healthcare.

#	Author	Year	Country	Study Setting	Aim	Design	Data collection tool/method	Participants	Predominant Decision-maker
1	Khemani, M. et al.	2020	India	Tertiary Care Teaching Institute	To assess the pathways of care and delay in seeking care for those individuals newly diagnosed with severe mental disorders (acute psychosis, depression, bipolar disorder (BD), and schizophrenia) at a tertiary teaching hospital in Puducherry in the southern part of India.	Cross sectional	Structured questionnaire	150 service users	Caregivers
2	Lilhare, V et al	2020	India	Home	To investigate the explanatory models (EMs) about mental illnesses and treatment-seeking behaviour (TSB) prevalent among the patients’ caregivers in Jharkhand, India		Semi-structured interviews exploring explanatory models (EMs)	240 caregivers	Caregivers
3	Aghukwa, CN et al	2012	Nigeria	Hospital	To examine treatment-seeking behaviour by 219 psychiatric patients at a teaching hospital in Kano	Cross sectional	Structured questionnaire with scales	219 service users and family members	Caregivers
4	Gomez, D M et al	2017	Sri Lanka; Hospital	Hospital	To study help-seeking behaviour and its impact on patients attending a psychiatry clinic at the National Hospital of Sri Lanka	Cross sectional	Structured questionnaire	120 service users	Caregivers
5	Gupta, A. et al	2021	Nepal	Hospital	To trace the various pathways and carers involved in mental healthcare; assess clinical variables such as the duration of untreated illness, clinical presentation, and treatment; 6 and compare geographically and culturally diverse landscapes.	Cross sectional	Pathways to Care survey	489 service users	Caregivers
6	Ismail, M.A et al	2021	Malaysia	Hospital	To determine the level of Shared Decision-Making and role preference and their associated factors among patients with schizophrenia in Malaysia	Cross sectional	Validated Shared Decision-Making Scale and structured questions	86 service users	Shared
7	Kotzé, C.; R et al	2021	South Africa	Hospital	To assess the end-of-life decision-making capacity and health-related values of older people with serious mental illness.	Cross sectional	Structured interviews	100 service users	Shared
8	Kudi, S.R.; Khakha, D.C.; Ajesh Kumar,	2022	India	Clinic	To identify pathways used by patients with severe mental disorders who seek mental healthcare services in the psychiatry unit of AIIMS, New Delhi	Cross sectional	Pathways to Care survey	123 caregivers	Caregivers
9	Lahariya, C et al	2010	India	Hospital	To understand the pathways of care adopted by the patients attending this facility, and to explore the interrelationship between the pathways of care and the socio-demographic variables	Cross sectional	Structured Pathways to Care questionnaire	295 service users	Caregivers
10	Odinka, Paul C	2014	Nigeria	Hospital	To determine the socio-demographic characteristics associated with treatment delay in people diagnosed with schizophrenia.	Cross sectional	Structured questionnaire	367 service users	Caregivers
11	Rai, N et al	2018	Nepal	Hospital	To provide descriptive insight into the pathways to care among the psychiatric patients attending the tertiary care hospital in Kathmandu Valley.	Cross sectional	Pathways to Care questionnaire	66 service users	Caregivers
12	Tarabey, L	2018	Lebanon	Psychiatric centres	To determine the assimilation of the concept of informed consent in psychiatric care and identify the obstacles that might hinder care	Cross sectional	Structured questionnaire	35 psychiatrists	Shared
13	Abate, A.W et al	2023	Ethiopia	Hospital	To examine the level and factors associated with shared decision-making practice in low-income countries like Ethiopia	Cross sectional	Shared Decision-Making Scale and structured questionnaire	423 service users	Shared
14	Gupta, A.K et al	2021	Nepal	Hospital	To evaluate the pathways to care, supernatural beliefs in caregivers, and duration of untreated psychosis.	Cross sectional	Pathways tool with structured questions,	133 service users and caregivers	Caregivers
15	Gyimah, L. et al	2023	Ghana	Prayer Camp	To explore the experiences of persons with mental illness receiving care in a faith-based setting in Ghana	Mixed Method	Qualitative interviews or focus groups	41 service users	Caregivers
16	Mathews, M et al	2019	India	Hospital	To explore the diversity of explanatory models among family caregivers at a centre for recovery-oriented rehabilitation services in South India.	Qualitative	Semi-structured interviews	60 family members	Caregivers
17	Barrow, L.F.m et al	2022	Gambia	Hospital	To describe local concepts, experiences, and knowledge about mental illness and the implications of such beliefs and attitudes for access to mental health services	Qualitative	Focus groups/interviews	15 service users and family members (specific breakdown not reported separately)	Caregivers
18	Mishra, N et al	2011	India	Clinic	To investigate various healthcare facilities used by a group of patients with mental health problems and reasons for using the services.	Qualitative	Structured and semi-structured questionnaire	200 service users	Caregivers
19	Dehbozorgi, R	2022	Iran	Clinic	To assess the barriers to family involvement in the care of patients with chronic mental illnesses	Qualitative	Structured interviews	34 healthcare providers, service users, and caregivers	Caregivers
20	Grover, S et al	2014	India	Hospital	To evaluate the relationship between the magico-religious beliefs held by the primary caregivers of patients with schizophrenia and first treatment contact pathways to care.	Qualitative	Belief scale plus structured questions	122 service users	Caregivers
21	Gurung, D. et al	2017	Nepal	Homes and health facilities	To explore (a) the extent and experiences of service user and caregiver involvement in policy-making, service planning, monitoring, and research in Nepal; (b) the perceived barriers to such involvement; and (c) possible strategies to overcome barriers	Qualitative	In-depth interviews and focus groups	24 service users and caregivers	Shared
22	Sichimba, F et al	2022	Zambia; Hospital	Hospital	To explore the cultural beliefs and practices surrounding mental illness as experienced by family caregivers caring for a next-of-kin with mental illness	Qualitative	Structured interviews	15 family members	Caregivers
23	Souraya, S et al	2018	Ethiopia	Healthcare Centre	To explore the involvement of people with schizophrenia in decision-making relating to their care in rural Ethiopia	Qualitative	Semi-structured interviews	28 service users	Shared
24	Banerjee, B. and Dixit, S.	2017	India	Clinic	To understand the explanatory models of the family members of persons suffering from common as well as severe mental illness	Qualitative	Semi-structured interviews	82 family members and 5 users	Caregivers
25	Abekah-Abekah-Carter, K, K.; Ayim, M.; Cudjoe, E.; Abdullah, A.; Chiu,	2024	Ghana	Home/ Health centre	To explore perspectives of twenty mental health professionals on kinship care for persons with severe and persistent mental illness in Ghana	Qualitative	Interviews and observation	20 mental health professionals	Caregivers/Health Professionals
26	Bahari, K.; Bachtiar, A.; Pertiwi, G.H.; Syahlani, A.	2024	Indonesia	Home	To investigate the burdens of these family caregivers. This descriptive phenomenological study involved 15 family caregivers who looked after persons with mental health conditionsin Malang City, Indonesia	Qualitative (Phenomenological)	Interviews and observation	15 family caregivers	Family caregivers
27	Asgari M, Adib M, Dehghan Nayeri N, Rezayat F.	2023	Iran	Hospital	To explore family caregivers’ experiences of the barriers to caregiving to patients with schizophrenia.	Qualitative	Semi-structured interviews	37 family caregivers	Family caregivers
28	Sakwape, K. et al	2025	Botswana	Home/ Health centre	To explore and describe the needs of family caregivers of people living with mental illness in Lobatse.	Qualitative	In-depth interviews	15 family caregivers	Family caregivers
29	Rapiya, B.; Khuzwayo, N.; Asher, L.; Brooke-Sumner, C.	2025	South Africa	Healthcare Centre	To explore the lived experiences, needs and coping strategies of caregivers with a family member with SMHC in a low-resource setting in South Africa, and their perspectives on the provision of a support group programme.	Qualitative	Interviews	15 family caregivers	Family caregivers and patients
30	Phoeun, B et al	2023	Cambodia	Home/ Community	To study caregiver burden to inform potential intervention approaches among relative caregivers of treatment-engaged mentally ill patients in Cambodia.	Qualitative	Focus groups	37 family caregivers	Family caregivers and patients
31	Luitel, N.P.; Lamichhane, B.; et al.	2025	Nepal	Home/ Health centre/ Community	To examine the factors that facilitate the initiation and continuation of treatment for depression or anxiety in Nepal.	Qualitative	McGill Illness Questionnaire	24 patients	Patients and family
32	Marutani, T.; Chhim, S.; Taing, S.; Nishio, A.	2025	Cambodia	Hospital	To examine the quality of life and its social determinants for patients with schizophrenia and family caregivers in Cambodia	Cross sectional	Structured Quality of Life Questionnaire	59 patients 59 caregivers	Family caregivers
33	Reddy, S.K.; Thirthalli, J et al.	2024	South India	Healthcare Centre	To explore the factors that prevented persons with schizophrenia and their caregivers from accessing psychiatric treatment using an interview schedule specifically developed for this purpose.	Qualitative	Validation Tool - Schedule of Factors Influencing Access to Treatment	67 persons with psychosis and family members	Patients and family caregivers
34	Jianfeng Zhu	2018	China	Home/ Health centre	To investigate how the political culture of Guan (care/control) in China is played out across the platforms of the state, the community and the family through the lens of caring for people with severe mental illness in urban Shanghai.	Qualitative	Observation and Interviews	2 patients and 2 families	Community and Family
35	Demoubly Kokota,1* Robert C. Stewart,2*	2023	Malawi	Literature/ Health centre	To understand pathways to care for psychosis in Malawi, is based on the Wellcome Trust Psychosis Flagship Report on the Landscape of Mental Health Services for Psychosis in Malawi.	Qualitative	Literature review and interviews	10 mental health experts	Patients, caregivers,
36	Zreik, T et al	2025	Lebanon	Home/ Community	To investigate barriers, facilitators, and power dynamics influencing service user participation at the micro-, meso- (service), and macro- (policy) levels.	Qualitative	Interviews and Focus groups	16 service users, 3 NGO staff, 3 UN Staff	Patients, families, clinicians

While the included studies had diverse populations and objectives, they generally addressed three overarching groupings related to decision-making in mental healthcare: active user involvement, family dominance, and drivers of decision-making. Each category captures different dimensions of what is known about how decisions are made in mental health contexts across various LMICs, as expanded below.

### Active user involvement

Eight studies discussed the extent of service users’ involvement in mental healthcare decisions, ranging from shared decision-making to participation in end-of-life choices. Two studies specifically examined shared decision-making through structured patient interviews [30,31]. In Malaysia, Ismail [30] found a significant mismatch between individuals’ desire for autonomy and their actual experience in care. Only 22% of those preferring autonomy had high scores (above 75) on the shared decision-making scale, while 65% experienced lower-than-expected engagement [32]. This reflected a systemic shortfall in aligning services with patients’ preferences. In Ethiopia, Abate [27] reported that user involvement in shared decision-making was positively associated with stronger social support and access to community-based health insurance. The study further found that lower perceived compassionate care, poor social support, and lack of community-based health insurance were associated with lower involvement in shared decision-making among people with mental illness.

Gurung [33] found that while users were present in forums, their participation was largely symbolic. Souraya [25], focusing on people with schizophrenia, found that involvement was typically delayed until patients were clinically stable. Interactions between caregivers and healthcare professionals often reinforced hierarchical structures, which constrained patients’ agency and perpetuated existing power imbalances.

In the Lebanese context, Tarabey’s study [34] found that family members - particularly close relatives such as parents, siblings, or spouses - often acted as surrogates in providing consent for psychiatric treatment. However, this arrangement was informal and not codified as surrogate in medical records. As a result, health professionals typically accepted their consent without systematically verifying its legal basis. This underscores the influential role of kinship ties in shaping informed consent practices in Lebanese psychiatric care.

In South Africa, Kotze [35] reported that individuals diagnosed with serious mental illness could be actively involved in end-of-life care decisions, with 58% demonstrating meaningful participation. Others, though lacking full decision-making capacity, still expressed preferences with support, thus reinforcing that capacity and participation exist on a life spectrum. This aligns with the concept of participatory or inclusive decision-making [36].

The Lebanese study by Zriek [37] provides the clearest articulation of what participants themselves considered meaningful involvement in that context. Examples included participation in treatment and recovery planning, being informed about available therapy options, having the ability to choose whether to continue with a particular provider, and engaging through community forums and feedback mechanisms. These practices were most reported in hospital settings. However, even when such practices were framed by providers as evidence of participation, key biomedical decisions, particularly medication, generally remained clinician-led.

In Nepal, Luitel [38] documented instances of self-initiated decision-making, especially among users with higher awareness about mental health care or exposure to information. Access to mental health literacy initiatives and prior positive engagement with services enabled some users to make autonomous decisions regarding when and where to seek care, which providers to consult, and whether to re-engage with services.

Across Lebanon and Nepal [37,38], a minority of users expressed full decisional confidence, asserting personal responsibility for decisions related to their mental health and reporting little reliance on family members or service providers. These cases, however, were exceptions rather than representative of dominant patterns of substitute decision-making regimes.

### Family dominance in decision-making

Across various LMICs, families were identified as predominant decision-makers in mental health contexts. Cultural norms and systemic gaps, such as under-resourced services, often compelled families to assume central roles, both by necessity and by tradition. This phenomenon is particularly rooted in communitarian values, where decisions are guided by collective rather than individualist frameworks [11]. Two interrelated roles emerged in the analysis: families as primary decision-makers and families as coordinators of care and support.

**a) Families as Primary Decision-Makers**

Across the reviewed studies, families emerged as pivotal actors in shaping mental healthcare decision-making. Multiple studies illustrated families’ central roles in determining when and where to seek care, selecting treatment pathways, choosing service providers, deciding on medications, and making medical appointments [25,39–43]. In some cases, family members provided consent on behalf of service users, especially where an individual’s decision-making capacity was questioned [34]. Family authority also extended to administering medications and identifying alternative healthcare providers.

Consistent evidence from Iran [44], Indonesia [45], Cambodia [46,47], Ghana [48], Malawi [49], South Africa [50], and Botswana [51] demonstrates that families frequently acted as proxy decision-makers. This was also evident in other studies from Ghana, Malaysia and Nigeria [30,52,53]. These indicated that during acute episodes, when service users were often described as having limited capacity to be involved in decisions about their own care, family members became proxy decision-makers.

Family dominance in decision-making was frequently legitimised through cultural, spiritual, and moral explanatory models of mental illness, including beliefs in witchcraft or moral transgression in Ghana and Nigeria [52,53]. Such interpretations often justified unilateral family action, including placement in prayer camps or seeking care from traditional healers as detailed below. In Indonesia and Cambodia [45,46], families commonly initiated care through religious or spiritual pathways - turning to biomedical services only when symptoms persisted. In these contexts, families also expressed that persons with mental illness lacked the competence to make informed decisions and required protection, framing substitute decision-making as benevolent.

In Ghana and Nigeria, family influence over decision-making was described as both overt and subtle, shaped by cultural expectations of obedience and collective responsibility [52,53]. In Ghana, stigma and fear of social disgrace further intensified family control, with decisions made aimed at concealment rather than recovery. As Gyimah [52] describes, caregivers responded to fears of shame by selecting prayer camps far from their communities and restricting visitors, limiting social exposure and autonomy. Similarly, people with severe mental illness were described as excluded from major household decisions and treated as if they had no capacity, legitimising substitute decision-making and reinforcing exclusion from both healthcare and everyday life [52,53].

Together, these findings highlight the layered intersections of family agency, community influence, cultural norms, stigma, and power in shaping decision-making. Families are commonly positioned as primary decision-makers, de-emphasising and sometimes ignoring the voices and preferences of people with mental illness.

**b) Families as Care Coordinators and Support Systems**

Beyond acting as primary decision-makers, families played a central role in coordinating care and providing ongoing support to people with mental illness. Across multiple settings, this form of involvement was often framed positively, reflecting caregiving responsibilities and support roles assumed by families in contexts of limited formal health service care [25,39,54,55]. Barrow [39] noted that families were responsible for coordinating care and providing day-to-day support. This ranged from involvement in treatment decisions to ensuring medication adherence.

In Iran, Dehbozorgi [54] described a family-centred care model in which families were recognised as experts in the treatment process. In this model, families collaborated closely with healthcare teams and were positioned as partners in care provision. Similarly, Souraya [25], in the study that was conducted in rural Ethiopia within a primary healthcare mental health integration context, reported that caregivers perceived themselves as having a legitimate right to make decisions on behalf of the service user due to the emotional, financial, and social burdens they carried. In Banerjee’s study [55], caregivers were not only actively involved in decision-making but were also viewed by health professionals as legitimate representatives of the patient. Notably, across these studies, there was little reference to opportunities for users to assert their own preferences or to participate meaningfully in decisions about their care.

In addition to decisional roles, families functioned as de facto care coordinators, particularly in overstretched health systems. They supervised medication adherence [48,50], coordinated appointments and transport [38,46], and managed daily care needs such as bathing, feeding, and clothing. Families also frequently acted as intermediaries between service users and healthcare providers, receiving clinical information directly while users remained excluded from communication.

While these roles ensured continuity of care and practical support, they often reinforced patterns of exclusion from decision-making. Authority and information tended to flow between professionals and families, positioning the service user as a passive recipient of care rather than an active participant.

### Drivers influencing decision-making

We identified three drivers that influenced decision-making in mental healthcare: belief systems and cultural norms, access to mental health services, and socioeconomic factors.

**a) Belief Systems and Cultural Norms**

Eight studies reported that explanatory beliefs about the causes of mental illness influenced care-seeking decisions [35,36,39,41,42,45,52,53]. Although these studies did not explicitly examine who ultimately made treatment decisions, they demonstrate how co-existing supernatural and biomedical explanations shaped the context within which individuals and families made decisions on care options.

In The Gambia, mental illness was commonly attributed to witchcraft or spirit possession, leading families to first consult traditional healers [35]. Similar findings were reported in Nigeria, where spiritual and moral explanations, including punishment for wrongdoing and spiritual attacks, encouraged families to pursue faith-based and traditional remedies before seeking psychiatric care [36]. In Zambia, caregivers frequently associated mental illness with spiritual possession or the neglect of ancestral rituals, viewing traditional healers, spiritual practitioners, and community elders as best equipped to address these perceived causes [39]. These beliefs, together with concerns about shame and stigma, also influenced family decision-making by prioritising the protection of family reputation over patient involvement in treatment choices [39,56]. In Sri Lanka, patients often sought assistance from religious leaders or alternative healers because mental illness was understood as having spiritual or karmic origins, with mental health services generally accessed only after these options had been exhausted [52].

Similarly, Gupta [53] reported that caregivers’ beliefs in celestial influences and evil spirits increased the likelihood of seeking non-medical forms of care. Comparable explanatory models were identified in Indonesia [41], Malawi [45], and Cambodia [42], where mental illness was commonly attributed to witchcraft, evil spirits, or divine punishment. These interpretations shaped care pathways by justifying the use of traditional healing, prayer, seclusion, or restraint in preference to, or before, biomedical mental health services.

Wider community norms also notably shaped decision-making processes. Gymah in Ghana [52] observed that decisions by people with schizophrenia or their family caregivers to pursue traditional treatments subject to influence exerted by relatives, the local community, and religious leaders. Tarabey [34] explored reasons for non-consenting for treatment in a clinical trial of both users and family members. They found fear of public disclosure emerging as a significant deterrent to involvement in decision-making. In other cases, families worried that visible participation in the trial would lead to assumptions about the worsening of the mental illness. Kinship norms in Ghana [48] and Nepal [38], further legitimised family authority over individual autonomy, prioritising collective responsibility and social order over personal choice. Stigma and fear of disgrace, reported in Iran, Ghana and Indonesia [44,45,48], influenced families to hide mental illness, restrict user behaviour, and limit visibility in order to protect family reputation.

**b) Access to Mental Health Services**

Four studies [33,39,54,57] found that limited access to mental health services, including geographical barriers, financial constraints, and scarcity of healthcare facilities, presented significant challenges for those seeking care. In these studies, families often struggled to access biomedical care, complicating decision-making. In The Gambia, Barrow [39] found that limited-service availability and stigma led many families to seek help from traditional or spiritual healers, but noted that families would prefer formal services if they were more accessible and trusted. Similarly, Gupta [57] observed that while supernatural models of understanding initially directed Nepali patients toward shamans, continued engagement with mental health services occurred once care was responsive and respectful. Dehbozorgi [54] further highlighted that non-availability of services discouraged continued use of formal care, whereas active inclusion of families improved adherence and trust. Gurung [38] reinforced this, showing that meaningful participation of service users and caregivers in Nepal fostered greater confidence in formal services and reduced uptake of informal pathways.

Structural barriers played a significant role in constraining decision-making. Distance, cost, and limited availability of services in South India Cambodia, Lebanon, and Nepal [37,38,47,58], restricted the ability of users and families to seek care, maintain continuity, and participate meaningfully in decisions about service use. Consequently, families and users often made constrained choices, opting for nearby services, discontinuing treatment prematurely, or relying on informal and alternative care pathways. In Lebanon [37], providers sometimes minimised the impact of these barriers, revealing a disconnect between service-user realities and professional assumptions.

**c) Social and Economic Factors**

Six studies identified socioeconomic status, social stigma, and religious beliefs as significant factors influencing decision-making processes in mental healthcare [25,29,30,35,38,43]. Financial constraints, lack of awareness, and stigma associated with mental illness strongly dictated help-seeking behaviors and treatment decisions [59]. For instance, in India, 71% of 150 individuals interviewed sought traditional healing due to its accessibility and affordability, with the majority coming from rural areas and being unemployed [41]. Souraya [25] further observed that when families were under financial constraints, wider family or community members were sometimes involved in supporting help-seeking and thereby influenced the decisions made to access care. Gurung [33] emphasized that limited education, low awareness, and challenging economic conditions hindered both users and caregivers from participating in decisions aimed at strengthening the health system.

Socioeconomic status also emerged as a central determinant of decisional power. Poverty and low educational attainment in Lebanon, Cambodia, Iran, and South India [37,44,47,58] were associated with users being labelled as passive or shy, reduced confidence to participate, and increased reliance on families or professionals to make decisions. In Lebanon [37], homelessness and precarious housing sharply reduced users’ influence over treatment decisions. Financial strain forced families into what were described as impossible choices, particularly in Cambodia [47], where decisions such as selling assets or discontinuing care were driven by survival needs rather than supporting treatment.

## Discussion

In this scoping review, we sought to map the extent and range of research on user and carer involvement in decision-making processes within mental healthcare in LMIC settings. This review highlights that mental healthcare decision-making in LMICs is shaped by complex interactions between individual agency, family relationships, cultural norms, and structural constraints. In the studies reviewed, the findings suggest that in many LMICs, decision-making is more about negotiations within families and communities rather than being made in isolation. This aligns with the idea of relational autonomy. This suggests that people make decsions within a web of social and institutional relationships, not just on their own. So, when we think about how service users get involved, it is not only about them as individuals. This perspective is consistent with the realities of many LMIC settings but also exposes tensions with the aims of the UNCRPD, which promotes supported decision-making and the preservation of legal capacity [1]. The challenge is therefore not to diminish the role of families, nor to frame these as necessarily obliged to follow UN aspirations, but to strengthen approaches that enable people with mental health conditions to exercise autonomy within these existing social relationships.

The review also demonstrates that meaningful involvement extends beyond clinical encounters. Participation is shaped by wider social and health system factors, including mental health literacy, service accessibility, poverty, and stigma. These contextual influences suggest that achieving meaningful engagement, as envisaged by the WHO Framework for Meaningful Engagement [2], will require health system strengthening alongside interventions that empower individuals, families and communities. Efforts to improve participation are therefore unlikely to succeed if they focus exclusively on clinician–patient interactions while overlooking the broader social determinants shaping decision-making.

An important finding is the limited representation of service-user perspectives within the evidence base. Although this review focused on involvement in decision-making, relatively few studies privileged first-person accounts, with much of the available evidence generated from caregiver or provider perspectives. This highlights an important gap in LMIC mental health research and suggests that current understandings of participation remain largely interpreted through others rather than through the lived experiences of people with mental health conditions themselves.

The review also points to important implications for policy and practice. Mental health systems should move beyond viewing family involvement and user participation as competing priorities and instead develop supported decision-making models that recognise the positive role of families while protecting the rights, preferences and autonomy of service users. Investing in service-user organisations, and training health professionals in shared and supported decision-making (and resourcing them to implement this), may help bridge the gap between current practice and international commitments to rights-based care [1,2].

There remains limited evidence on how supported decision-making can be implemented in resource-constrained settings, how involvement varies across different mental health conditions, and how decision-making evolves over time. Greater attention is also needed to evaluating interventions that strengthen meaningful participation within culturally diverse and family-oriented contexts and also focusing on specific mental health conditions. Addressing these gaps will be essential for informing policies and practices that promote autonomy while recognising the relational realities of mental healthcare and structural constraints on decision-making in LMICs

### Recommendations for future research

Future research should prioritise the development and evaluation of culturally appropriate models of supported decision-making that balance individual autonomy with the important role of families in LMICs. Greater use of co-produced and participatory research is needed to ensure that people with mental health conditions actively shape research, policy and practice rather than remaining subjects of investigation. Longitudinal and implementation studies are also required to examine how meaningful involvement can be strengthened across different care settings and stages of recovery. Finally, comparative studies across LMICs would help identify context-specific and transferable strategies for reducing structural barriers, strengthening mental health systems, and promoting rights-based, person-centred decision-making in line with the UNCRPD and the WHO Framework for Meaningful Engagement.

### Limitations

This scoping review has several limitations. First, despite including both peer-reviewed and grey literature, relevant evidence contained in books, monographs, reports, and unpublished materials may have been missed. Second, only English-language publications were included, potentially excluding relevant studies from regions where other languages predominate and limiting the global representativeness of the findings. Third, the review included studies employing diverse methodologies, settings, participant groups, and definitions of mental health conditions. While such heterogeneity is expected in scoping reviews and allowed a broad mapping of the evidence base, it limits direct comparability across studies. Finally, because the review focused on publications from 2008 onwards, it does not capture earlier conceptualisations of involvement in mental healthcare decision-making prior to the implementation of the UNCRPD.

## Conclusion

This scoping review demonstrates that user and caregiver involvement in mental healthcare decision-making within LMICs is shaped by complex interactions between family relationships, cultural expectations, socioeconomic conditions, and health system structures. While examples of active participation and shared decision-making are emerging, family-led and substitute forms of decision-making continue to predominate across many contexts. Viewed through the lenses of the UNCRPD and the WHO Meaningful Engagement Framework, the findings reveal substantial gaps between current practice and international commitments to rights-based and participatory care.

Strengthening involvement will require more than individual-level interventions. It will necessitate investments in enabling supported decision-making approaches, alongside investing in service-user organisations and accessible services, and the development of policy frameworks that institutionalise participation. Future research should pay greater attention to contextual variations across settings, diagnoses, and care pathways, while exploring how users, families, and professionals negotiate decisions in everyday practice. Such work will be essential to advancing autonomy, dignity, and meaningful participation in mental healthcare across LMICs.

## Supporting information

S1 Appendix Scoping Review Search Strategy. (DOCX)

S1 PRISMA Checklist PRISMA ScR Checklist. (DOCX)
