# Supplementary material for: Involvement of users and caregivers in mental healthcare decision-making within low-and middle-income countries: A scoping review
Source: PLOS Ment Health. 2026 Oct 30;3(10):e0000712. doi: 10.1371/journal.pmen.0000712 (PMC13722329; doi:10.1371/journal.pmen.0000712)
Supplement: Supplementary file 1 [file pmen.0000712.s001.docx]

PUBMED (MEDLINE)

#1 Service users / carers

("patient participation"[MeSH Terms] OR "caregivers"[MeSH Terms] OR caregiver*[tiab] OR "family"[tiab] OR carer*[tiab] OR service user*[tiab] OR client*[tiab] OR consumer*[tiab] OR "persons with mental illness"[tiab])

#2 Involvement / engagement

("decision making"[MeSH Terms] OR "shared decision making"[MeSH Terms] OR "patient participation"[MeSH Terms] OR participat*[tiab] OR involv*[tiab] OR engag*[tiab] OR empower*[tiab] OR collaborat*[tiab])

#3 Mental health

("mental health"[MeSH Terms] OR "mental disorders"[MeSH Terms] OR psychiatr*[tiab] OR "psychosocial"[tiab] OR "psychological"[tiab] OR "mental illness"[tiab] OR depression[tiab] OR schizophrenia[tiab] OR bipolar[tiab])

#4 Combine

#1 AND #2 AND #3

#5 LMIC filter

#4 AND (("Developing Countries"[MeSH]) OR (low income countr*[tiab] OR middle income countr*[tiab] OR Africa[tiab] OR Asia[tiab] OR "Latin America"[tiab] OR Caribbean[tiab] OR "Pacific Islands"[tiab]))

PUBMED VIA MEDLINE

(

("Caregivers"[Mesh] OR "Family"[Mesh] OR "Patient Participation"[Mesh]

OR caregiver*[tiab] OR carer*[tiab] OR family[tiab] OR relative*[tiab]

OR "service user*"[tiab] OR "client*"[tiab] OR consumer*[tiab] OR "persons with mental illness"[tiab])

)

AND

(

("Decision Making"[Mesh] OR "Shared Decision Making"[Mesh] OR "Patient Participation"[Mesh]

OR participat*[tiab] OR involv*[tiab] OR engag*[tiab] OR empower*[tiab] OR collaborat*[tiab])

)

AND

(

("Mental Health"[Mesh] OR "Mental Disorders"[Mesh]

OR psychiatr*[tiab] OR "mental illness"[tiab] OR "mental health"[tiab]

OR psychosocial[tiab] OR depression[tiab] OR schizophrenia[tiab] OR bipolar[tiab])

)

AND

(

("Developing Countries"[Mesh] OR "low income countr*"[tiab] OR "middle income countr*"[tiab]

OR Africa[tiab] OR Asia[tiab] OR "Latin America"[tiab] OR Caribbean[tiab] OR "Pacific Islands"[tiab])

EMBED via OVID and Elsevier

(

'caregiver'/exp OR 'family'/exp OR 'patient participation'/exp

OR caregiver*:ti,ab OR carer*:ti,ab OR family:ti,ab OR relative*:ti,ab

OR 'service user*':ti,ab OR client*:ti,ab OR consumer*:ti,ab OR 'persons with mental illness':ti,ab

)

AND

(

'decision making'/exp OR 'shared decision making'/exp OR 'patient participation'/exp

OR participat*:ti,ab OR involv*:ti,ab OR engag*:ti,ab OR empower*:ti,ab OR collaborat*:ti,ab

)

AND

(

'mental health'/exp OR 'mental disease'/exp

OR psychiatr*:ti,ab OR 'mental illness':ti,ab OR 'mental health':ti,ab

OR psychosocial:ti,ab OR depression:ti,ab OR schizophrenia:ti,ab OR bipolar:ti,ab

)

**PsycINFO (APA Thesaurus + free text; EBSCO example)**

(

DE "Caregivers" OR DE "Family" OR DE "Patient Participation"

OR caregiver* OR carer* OR family OR relative*

OR "service user*" OR client* OR consumer* OR "persons with mental illness"

)

AND

(

DE "Decision Making" OR DE "Shared Decision Making" OR DE "Patient Participation"

OR participat* OR involv* OR engag* OR empower* OR collaborat*

)

AND

(

DE "Mental Health" OR DE "Mental Disorders" OR DE "Psychiatry"

OR psychiatr* OR "mental illness" OR "mental health" OR psychosocial

OR depression OR schizophrenia OR bipolar

)

**Scopus (Elsevier, TITLE-ABS-KEY)**

TITLE-ABS-KEY(

(caregiver* OR carer* OR family OR relative* OR "service user*" OR client* OR consumer* OR "persons with mental illness")

AND

(participat* OR involv* OR engag* OR empower* OR collaborat* OR "decision making" OR "shared decision making")

AND

(psychiatr* OR "mental illness" OR "mental health" OR psychosocial OR depression OR schizophrenia OR bipolar)

)

**Web of Science (Core Collection, TS field)**

TS=(

(caregiver* OR carer* OR family OR relative* OR "service user*" OR client* OR consumer* OR "persons with mental illness")

AND

(participat* OR involv* OR engag* OR empower* OR collaborat* OR "decision making" OR "shared decision making")

AND

(psychiatr* OR "mental illness" OR "mental health" OR psychosocial OR depression OR schizophrenia OR bipolar)

)
