# Supplementary material for: Involvement of users and caregivers in mental healthcare decision-making within low-and middle-income countries: A scoping review
Source: PLOS Ment Health. 2026 Oct 30;3(10):e0000712. doi: 10.1371/journal.pmen.0000712 (PMC13722329; doi:10.1371/journal.pmen.0000712)
Supplement: Supplementary file 2 [file pmen.0000712.s002.docx]

**Preferred Reporting Items for Systematic reviews and Meta-Analyses extension for Scoping Reviews (PRISMA-ScR) Checklist**

| **SECTION** | **ITEM** | **PRISMA-ScR CHECKLIST ITEM** | **REPORTED ON PAGE #** |
| --- | --- | --- | --- |
| **TITLE** | | | |
| Title | 1 | The title clearly identifies the report as a scoping review: *“Involvement of users and caregivers in mental healthcare decision-making within low- and middle-income countries: a scoping review.”* | 1 |
| **ABSTRACT** | | | |
| Structured summary | 2 | Provide a structured summary that includes (as applicable): background, objectives, eligibility criteria, sources of evidence, charting methods, results, and conclusions that relate to the review questions and objectives.  **Abstract**  **Background**  Inclusive healthcare decision-making is essential for recovery-oriented mental health services, as it improves outcomes and reduces stigma. Most studies on decision-making in mental healthcare have been conducted in high-income countries, leaving a significant gap in understanding how diverse sociocultural and health system contexts in low- and middle-income countries (LMICs) shape these processes. In many LMICs, decision-making often reflects communal values, emphasising the collective over the individual. There is limited evidence on the involvement of service users and caregivers in mental healthcare decision-making in LMICs.  **Objective**  This scoping review asked: how are service users and caregivers involved in mental healthcare decision-making in LMICs, and what contextual factors shape these processes? The review aimed to identify and collate studies contributing to the understanding of service users’ and caregivers’ involvement in mental healthcare decision-making within LMICs and to characterise salient contextual factors shaping such involvement.  **Methods**  Following Arksey and O’Malley’s five-stage scoping review framework, English-language publications from 2008 to 2025 were searched across multiple databases, with additional studies identified through grey literature searches. Eligible studies focused on service users and/or caregivers in LMICs and reported on involvement in mental healthcare decision-making. Data were charted and analysed using interpretative and content analysis to identify patterns of involvement as described in the included studies.  **Results**  The initial search yielded 21,830 records, with an additional 46 studies identified through grey literature searches. After screening, 36 studies were included. The included studies were conducted in Africa (n=12), Asia (n=22), and the Middle East (n=2). Findings were grouped into three areas: (1) active user involvement, (2) family dominance, and (3) drivers of decision-making. Active user involvement described instances where service users actively participated in or co-created decision-making processes, demonstrating agency in shaping care outcomes. Family dominance reflected situations where families took control of decisions, influencing or overriding service users’ preferences. Drivers of decision-making captured broader contextual and systemic influences, including cultural norms, economic challenges, stigma, and resource availability.  Conclusions  This review underscores the central role of family in mental healthcare decision-making in LMICs amid economic hardship and stigma. Limited attention has been given to contextualised conceptualisations of decision-making in LMICs, and important gaps remain in understanding how decisions are made, who influences them, and how these processes unfold over time.  Keywords: user involvement; carer involvement; mental healthcare; decision-making; LMICs | 2-3 |
| **INTRODUCTION** | | | |
| Rationale | 3 | Describe the rationale for the review in the context of what is already known. Explain why the review questions/objectives lend themselves to a scoping review approach.  The rationale is that involvement of people with lived experience in mental healthcare decision making is a human rights imperative under the UNCRPD and WHO meaningful engagement guidance, yet evidence from LMICs remains limited. Existing studies suggest that decision-making in LMICs is shaped by communitarian values, family and community roles, under-resourced systems, and the persistence of substitute decision-making. A scoping review was appropriate because the evidence base is broad, heterogeneous, and conceptually underdeveloped, and the review aimed to map the extent, range, and nature of studies, clarify how service users and caregivers are involved, identify contextual drivers, and highlight gaps for future research. | 4-5 |
| Objectives | 4 | Provide an explicit statement of the questions and objectives being addressed with reference to their key elements (e.g., population or participants, concepts, and context) or other relevant key elements used to conceptualize the review questions and/or objectives.  The review explicitly states the objective and research question: to identify, collate, and map literature on how persons identified as having mental disorders and caregivers in LMICs are involved in mental healthcare decision-making across home and healthcare (formal and informal) settings, and to characterise contextual factors shaping such involvement. The question was framed using the PCC framework: Population = persons with mental health conditions and caregivers in LMICs; Concept = involvement in mental healthcare decision-making; Context = home and formal/informal care settings, including traditional and faith healing sites | 6-7 |
| **METHODS** | | | |
| Protocol and registration | 5 | Indicate whether a review protocol exists; state if and where it can be accessed (e.g., a Web address); and if available, provide registration information, including the registration number.  A review protocol was developed and registered with the Open Science Framework (OSF) and is publicly accessible at <https://doi.org/10.17605/OSF.IO/WM2UX>. | 6 |
| Eligibility criteria | 6 | Specify characteristics of the sources of evidence used as eligibility criteria (e.g., years considered, language, and publication status), and provide a rationale.  Eligibility criteria included English-language studies (2008–2025) involving persons with mental health conditions and/or caregivers in LMICs and addressing involvement in mental healthcare decision-making across home and formal/informal care settings. The 2008 start date was justified by the entry into force of the UNCRPD. Eligibility criteria are clearly reported in Table 1, including concept/focus, population, context (LMICs) with rationale linked to the entry into force of the UNCRPD in 2008. Inclusion and exclusion criteria are explicitly stated. | 8 |
| Information sources* | 7 | Describe all information sources in the search (e.g., databases with dates of coverage and contact with authors to identify additional sources), as well as the date the most recent search was executed.  Information sources included Ovid MEDLINE, Embase, PsycINFO, Social Science Citation Index, Global Health, and SCOPUS, plus grey literature sources. Searches covered 2008–2025 and final search date for all databases was 25 September, 2025. All authors were contacted to verify any gaps in the search and worked with the first author. | 7 |
| Search | 8 | Present the full electronic search strategy for at least 1 database, including any limits used, such that it could be repeated.  The search strategy was developed by the lead reviewer in consultation with a specialist librarian and the wider review team. The full electronic search strategy for at least one database, including search terms, subject headings, Boolean operators, and limits used, is provided in Supporting Information Appendix I. Search date is 25 September, 2025 | 7 Appendix I |
| Selection of sources of evidence† | 9 | State the process for selecting sources of evidence (i.e., screening and eligibility) included in the scoping review.  Records were deduplicated and screened against predefined inclusion/exclusion criteria. Titles/abstracts were reviewed by three reviewers, disagreements were resolved through discussion and adjudication, and potentially eligible studies underwent full-text review. Final selection is shown in Figure 1. | 10 |
| Data charting process‡ | 10 | Describe the methods of charting data from the included sources of evidence (e.g., calibrated forms or forms that have been tested by the team before their use, and whether data charting was done independently or in duplicate) and any processes for obtaining and confirming data from investigators.  Data were charted using a standardised Excel data extraction sheet developed for the review. Two reviewers (AA and SJumbe) independently extracted/charted data. The study authors were contacted for clarification and testing the form | 9 |
| Data items | 11 | List and define all variables for which data were sought and any assumptions and simplifications made.  Data extracted included study characteristics, setting, participants, and descriptions of decision-making processes involving persons with mental disorders, forms of service user/caregiver involvement, and contextual factors shaping involvement. The manuscript also notes that explicit references to decision-making involvement were often absent, so decision-making processes were sometimes inferred from descriptions of actions, roles, interactions, and care pathways. | 13-31 |
| Critical appraisal of individual sources of evidence§ | 12 | If done, provide a rationale for conducting a critical appraisal of included sources of evidence; describe the methods used and how this information was used in any data synthesis (if appropriate).  No formal critical appraisal of included studies was undertaken, consistent with scoping review methodology and the aim of mapping the breadth and nature of evidence rather than assessing study quality. | N/A |
| Synthesis of results | 13 | Describe the methods of handling and summarizing the data that were charted.  Extracted data were organised in a standardised Excel charting sheet and synthesised descriptively using numerical summary and narrative/thematic synthesis to map study characteristics, forms of involvement, and contextual factors influencing service user and caregiver participation in mental healthcare decision-making in LMICs. The extracted data was systematically coded and categorised using content analysis, alongside an interpretative examination of how reported practices reflected underlying decision-making dynamics, including power relations and exclusion. This approach was used to summarise heterogeneous evidence base where decision making involvement was often implicit rather than explicitly named. | 14-31 |
| **RESULTS** | | | |
| Selection of sources of evidence | 14 | Give numbers of sources of evidence screened, assessed for eligibility, and included in the review, with reasons for exclusions at each stage, ideally using a flow diagram.  The results section reports the numbers of records identified, screened, excluded, and included. Specifically, 21,830 titles were screened, 46 additional studies were identified from other sources, 10,845 were excluded at stage one, 141 full texts were assessed, 105 were excluded at full-text stage, and 36 studies were included. A PRISMA flow diagram (Figure 1) is provided. | 10 |
| Characteristics of sources of evidence | 15 | For each source of evidence, present characteristics for which data were charted and provide the citations.  Characteristics of included studies are presented in the study summary table, including author/year, country/setting, study design, participant group(s), and decision-making/care context. Also the manuscript presents the characteristics of included studies, including regional distribution across Africa (n=12), Asia (n=22), and the Middle East (n=2), and describes the predominant countries, study focus areas, and study designs (e.g., qualitative studies, pathways to care, family involvement, stigma, power dynamics, and service-user participation). Citations to included studies are provided in the manuscript and reference list. | 14-31 |
| Critical appraisal within sources of evidence | 16 | If done, present data on critical appraisal of included sources of evidence (see item 12).  No formal critical appraisal was undertaken; therefore, no critical appraisal results are reported. This is consistent with the scoping review objective of mapping the breadth and nature of evidence rather than evaluating study quality | N/A |
| Results of individual sources of evidence | 17 | For each included source of evidence, present the relevant data that were charted that relate to the review questions and objectives.  Table 1 and Results section. Relevant findings from individual included studies are presented in the study summary table and narrative synthesis, including forms of service user/caregiver involvement in decision-making and contextual influences across care settings describing how individual studies contributed evidence on service-user involvement, family roles, pathways to care, power dynamics, and contextual influences on decision-making. | 9-14 |
| Synthesis of results | 18 | Summarize and/or present the charting results as they relate to the review questions and objectives.  Results are synthesized and summarises charted findings into three main analytical groupings: (1) active user involvement, (2) family dominance, and (3) drivers of decision-making. These categories are used to organise the evidence in relation to the review objectives and to show how agency, power, culture, stigma, economic hardship, and resource constraints shape mental healthcare decision-making in LMICs. | 9-42 |
| **DISCUSSION** | | | |
| Summary of evidence | 19 | Summarize the main results (including an overview of concepts, themes, and types of evidence available), link to the review questions and objectives, and consider the relevance to key groups.  The Discussion summarises the main findings in relation to the review objectives, highlighting that service user involvement in mental healthcare decision-making in LMICs is often limited and context-dependent, caregivers frequently play central roles, and involvement is shaped by family norms, informal care pathways, under-resourced systems, and the continued dominance of substitute over supported decision-making. It also highlights the importance of sociocultural and structural influences, and the relevance of these findings to service users, caregivers, clinicians, and policy actors. The discussion links the findings to the UNCRPD and WHO meaningful engagement framework. | 42-49 |
| Limitations | 20 | Discuss the limitations of the scoping review process.  The Discussion outlines limitations of the review process, including possible omission of relevant studies, heterogeneity of included studies and contexts, variability in reporting of decision-making involvement. The manuscript discusses limitations of evidence in research monographs may have been missed, the restriction to English-language publications, and the 2008 onward time limit, which excludes earlier conceptualisations of involvement in decision-making. | 49 |
| Conclusions | 21 | Provide a general interpretation of the results with respect to the review questions and objectives, as well as potential implications and/or next steps.  The conclusion provides an overall interpretation of the findings in relation to the review objectives, noting that user and carer involvement is shaped by family influence, cultural norms, structural systems, and socioeconomic constraints. It also highlights the need for future research and the implications for policy, clinical practice, and rights-based efforts to enhance autonomy in mental healthcare. | 49-50 |
| **FUNDING** | | | |
| Funding | 22 | Describe sources of funding for the included sources of evidence, as well as sources of funding for the scoping review. Describe the role of the funders of the scoping review.  The manuscript highlights funding for the scoping review, stating that the study was funded by the Wellcome Trust (grant 223615/Z/21/Z), and also discloses additional support for individual authors from NIHR, the British Academy, and Wellcome. | 51 |

JBI = Joanna Briggs Institute; PRISMA-ScR = Preferred Reporting Items for Systematic reviews and Meta-Analyses extension for Scoping Reviews.

* Where *sources of evidence* (see second footnote) are compiled from, such as bibliographic databases, social media platforms, and Web sites.

† A more inclusive/heterogeneous term used to account for the different types of evidence or data sources (e.g., quantitative and/or qualitative research, expert opinion, and policy documents) that may be eligible in a scoping review as opposed to only studies. This is not to be confused with *information sources* (see first footnote).

‡ The frameworks by Arksey and O’Malley (6) and Levac and colleagues (7) and the JBI guidance (4, 5) refer to the process of data extraction in a scoping review as data charting*.*

§ The process of systematically examining research evidence to assess its validity, results, and relevance before using it to inform a decision. This term is used for items 12 and 19 instead of "risk of bias" (which is more applicable to systematic reviews of interventions) to include and acknowledge the various sources of evidence that may be used in a scoping review (e.g., quantitative and/or qualitative research, expert opinion, and policy document).

*From:* Tricco AC, Lillie E, Zarin W, O'Brien KK, Colquhoun H, Levac D, et al. PRISMA Extension for Scoping Reviews (PRISMAScR): Checklist and Explanation. Ann Intern Med. 2018;169:467–473. [doi: 10.7326/M18-0850](http://annals.org/aim/fullarticle/2700389/prisma-extension-scoping-reviews-prisma-scr-checklist-explanation).
