# Supplementary material for: Involvement of users and caregivers in mental healthcare decision-making within low-and middle-income countries: A scoping review
Source: PLOS Ment Health. 2026 Oct 30;3(10):e0000712. doi: 10.1371/journal.pmen.0000712 (PMC13722329; doi:10.1371/journal.pmen.0000712)
Supplement: Supplementary file 3 [file pmen.0000712.s003.docx]

Date:

Rebuttal Letter to the Editor – PLOS Mental Health

Dear Dr. Beura and the Editorial Team at PLOS Mental Health,

Thank you for your previous assessment of our manuscript and for the constructive guidance provided regarding the standards expected for scoping reviews at PLOS Mental Health. We appreciate the opportunity to revise the work and resubmit it as a new submission, with reference to the original manuscript as advised.

We respectfully submit a substantially revised version of our scoping review manuscript and wish to indicate that the key concerns raised in the previous editorial decision have now been carefully addressed.

First, we have undertaken a fully updated and expanded literature search to ensure that the review provides a current and comprehensive overview of the evidence base. In response to the concern that the prior review was completed over a year ago, the search strategy has now been rerun and updated to include the most recent available literature up to 25^th^ September, 2025. This updated search was conducted across all relevant databases, and the revised manuscript now reflects newly identified studies where applicable. We believe this substantially strengthens the timeliness and relevance of the review.

Secondly, we have revised the methods section extensively to ensure that the review process is clearly reported, reproducible, systematic, and transparent. The manuscript now provides a more detailed description of the search strategy, databases searched, eligibility criteria, screening process, study selection procedures, data charting process, and approach to synthesis. We have also ensured closer alignment with established scoping review guidance, including the PRISMA-ScR framework, and have strengthened the reporting of the review workflow to improve clarity and methodological rigour.

In addition, we have reviewed the manuscript in light of the PLOS guidance for systematic and scoping reviews to ensure that the revised submission more fully meets journal expectations regarding transparency, comprehensiveness, and reproducibility. This includes strengthening the presentation of inclusion and exclusion criteria, improving documentation of study selection, and ensuring that the synthesis of findings is explicitly linked to the stated review question.

For ease of reference, the principal issues raised in the previous editorial decision and our responses are summarized below:

1. **Concern: The manuscript did not meet PLOS Mental Health standards for scoping reviews.**
   **Response:** The manuscript has been substantially revised to strengthen the scoping review design, reporting structure, and methodological transparency in line with accepted scoping review standards and journal guidance.
2. **Concern: The literature review was out of date.**
   **Response:** The search has now been updated through 25^th^ September, 2026, and the revised manuscript incorporates newly identified relevant studies to ensure the review reflects the current state of the evidence.

We are grateful for the feedback provided in the original decision, which has helped us substantially improve the manuscript. We believe that the revised submission is now more methodologically robust, up to date, and better aligned with the standards expected for scoping reviews at PLOS Mental Health.

We respectfully ask that the editorial team consider this revised manuscript as a new submission, as advised, and we hope it may now be suitable for peer review.

Thank you for your time and consideration.

Yours sincerely,

Action Amos
On behalf of all co-authors
