# Supplementary material for: Involvement of users and caregivers in mental healthcare decision-making within low-and middle-income countries: A scoping review
Source: PLOS Ment Health. 2026 Oct 30;3(10):e0000712. doi: 10.1371/journal.pmen.0000712 (PMC13722329; doi:10.1371/journal.pmen.0000712)
Supplement: Supplementary file 4 [file pmen.0000712.s004.docx]

**14^th^ August, 2026**

**The Editor**

**PLOS Mental Health**

**Subject: Response to Editor and Reviewers – Revised Manuscript**

Thank you for the opportunity to revise and resubmit our manuscript to PLOS Mental Health. We are grateful to you and the reviewers for the thoughtful, constructive, and detailed comments. The feedback has helped us strengthen the manuscript substantially.

We have carefully considered all comments and revised the manuscript accordingly (see Response Table below). In particular, we have strengthened the **Abstract and Introduction,** expanded the description of the **analytical and synthesis processes in the Methods, c**larified the conceptualisation of **involvement and the use of relational autonomy as a theoretical lens,** and revised the **Results and Discussion** to reduce repetition and strengthen the distinction between reporting findings and interpreting them.

We have also strengthened the practical and policy implications of the review, including greater attention to **caregiver participation** and the need for processes that support meaningful involvement in mental healthcare decision-making. We have further clarified the scope and interpretation of the included evidence, including the geographical concentration of studies, the limitations associated with English-language publications, variation in conceptualisations of mental health conditions, and the inclusion of studies involving healthcare professionals where these provided relevant insight into decision-making involvement.

The revised manuscript also provides greater clarity regarding the characteristics of the included studies and the synthesis process. We have strengthened the explanation of how the findings were interpreted through the lens of **relational autonomy**, highlighting how the findings demonstrate that decision-making is shaped not only by individual preferences and capacities but also by relationships, family and caregiver involvement, social circumstances, health-system factors, and broader structural conditions.

We sincerely appreciate the time, expertise, and constructive guidance provided by the Editor and reviewers. We believe that the revisions have substantially strengthened the manuscript and improved its clarity, theoretical contribution, methodological transparency, and relevance to mental health research and practice. We hope that the revised manuscript is now suitable for publication in PLOS Mental Health and thank you again for the opportunity to revise our work.

Yours sincerely,

**Action AMOS**
On behalf of all authors

**EDITOR COMMENTS**

| **Editor's Comment** | **Author Response** | **Location in Revised Manuscript** |
| --- | --- | --- |
| *I would be keen to see this paper include greater detail and thought on the implications of your review for practitioners and more broadly for practice. Please add this to your Discussion section.* | We sincerely thank you for this valuable recommendation. We agree that strengthening the practical implications of the review enhances its relevance for policymakers, practitioners, researchers, and service-user organisations. In response, we have added a paragraph to the Discussion. We believe these additions considerably strengthen the practical contribution of the manuscript and provide actionable recommendations for improving mental healthcare decision-making in low- and middle-income countries. | **Pages:46**  **Lines:551- 557** |
| *I am also wondering if your review explored how the included studies document the processes for participation of caregivers in decision making that are in place and working well (if there is evidence of this), and if so, I would like this to be more strongly reflected in both the findings and discussion in order to make this paper more useful to practitioners.* | We thank you for this query. We carefully revisited the included studies to examine whether they described processes that facilitate meaningful caregiver participation in mental healthcare decision-making. While the review identified several examples of practices including communication between healthcare professionals and families, no studies explicitly evaluated formal models of caregiver participation or assessed their effectiveness. However, the positive engagement approaches used by caregivers are highlighted in the results under the heading Families as Care Coordinators and Support Systems and is also highlighted in the Discussion. In our conclusion note that we have added the need for more understanding about processes to support participation in the conclusions | **Results**  **Pages:39**  **Lines:**377-405  **Discussion**  Page 45  Lines 532-538 |

**REVIEWER 1**

| **Reviewer's Comment** | **Author Response** | **Location in Revised Manuscript** |
| --- | --- | --- |
| *The abstract should include the following: purpose of the study, methodology used, findings, discussion, recommendations, and contributions. General background information needs to be provided with a description of the gap in our knowledge that the study was designed to fill. The methodology also needs to provide more detail on how the analysis was undertaken. There is excessive repetition throughout the manuscript, the discussion is overly lengthy, and some paragraphs merely restate the literature without deeper analysis.* | We thank you for this assessment. We have carefully revised the manuscript to address each of the issues raised. First, the **Abstract** has been substantially revised to provide a clearer and more structured summary of the study, including the background and knowledge gap, study objective, methodological approach, principal findings, implications for policy and practice, and the overall contribution of the review. The revised abstract remains consistent with the formatting requirements of **PLOS ONE** while presenting a more informative overview of the study. Second, the **Introduction** has been strengthened to more clearly articulate the knowledge gap that motivated the review, specifically the limited synthesis of evidence regarding the involvement of persons with mental health conditions and their caregivers in decision-making within LMICs. Third,  | **Abstract**  **Pages:**2-3 **Lines:**1-26 |
|  | the **Methods** section has been expanded to provide a more transparent description of the analytical process. We now describe how study findings were extracted, organised into an analytical matrix, independently coded using an inductive content analysis approach, grouped into higher-order categories through iterative discussion, and synthesised into overarching themes. | **Analysis**  **Pages:**2-3 **Lines** 18-26 |
|  | Finally, we have carefully revised the whole **Discussion** section to reduce repetition, improve the depth of interpretation, and ensure a clearer distinction between descriptive findings and critical analysis. These revisions have strengthened the overall coherence, readability, and contribution of the manuscript. | **Discussion**  **Pages:**45-46 **Lines:**500-546 |
| *The conclusion of the abstract should more clearly summarise the principal findings and implications of the review.* | We appreciate this recommendation and have revised the concluding section of the Abstract to more accurately reflect the principal findings and their implications. The revised conclusion now emphasises that mental healthcare decision-making in LMICs continues to be shaped by family authority, cultural norms, and structural constraints, with relatively limited evidence of supported or shared decision-making. The revised conclusion also clearly articulates the contribution of this review to policy, practice, and future research within LMIC settings. | **Pages:**3  **Lines:34-**38 |

**REVIEWER 2**

| **Reviewer's Comment** | **Author Response** | **Location in Revised Manuscript** |
| --- | --- | --- |
| *The selection of countries included in the analysis would benefit from further clarification, as this would strengthen the framing and contextual understanding of the findings.* | We thank the reviewer for this observation. We agree that explaining the geographical distribution of the included studies provides important context for interpreting the findings. In response, we have expanded the **Study Characteristics** section to discuss the uneven geographical distribution of the evidence. Based on the authors experiences we note that the concentration of studies from countries such as India, Nepal, Ethiopia, and Lebanon is likely to reflect differences in research capacity, funding availability, publication practices, language limitations and the maturity of national mental health research programmes rather than the true distribution of decision-making practices across LMICs. | **Pages:**15 **Lines:**238-243 |
| *Please provide greater detail regarding the analytical process used to synthesise the findings.* | We appreciate the reviewer's recommendation and agree that greater methodological transparency strengthens the credibility of the review. This is elaborated both in the methods section and in the Abstract. Accordingly, we have expanded the **Methods** section in the Abstract section to provide a more detailed description of the analytical approach. Specifically, we now explain that following data extraction, findings relating to decision-making involvement were organised into an analytical matrix. Two reviewers independently coded the extracted data using an inductive content analysis approach. Similar codes were grouped into higher-order categories through an iterative process of discussion and consensus, and these categories were subsequently synthesised into three overarching themes describing patterns of decision-making involvement across the included studies. This additional detail improves the transparency, rigour, and reproducibility of the analytical process. | **Abstract**  **Pages:**2-3 **Lines:**17-22  **Methods**  **Pages:**11-12 **Lines:**179-184 |

**REVIEWER 3**

| **Reviewer's Comment** | **Author Response** | **Location in Revised Manuscript** |
| --- | --- | --- |
| *While the methodology is robust and aligned with established frameworks, the study would benefit from deeper theoretical grounding, particularly in relation to power dynamics and relational autonomy. Overall, the paper makes an important contribution but would be enhanced by more critical analysis, broader inclusion, and stronger integration of theory and policy implications.* | Thank you for this helpful suggestion. We have clarified in the Methods that **relational autonomy** was used as the theoretical lens to guide the synthesis and interpretation of the included studies. We have also expanded the Discussion to demonstrate how the findings align with relational autonomy, showing that decision-making is shaped by social relationships, cultural contexts, and structural conditions, rather than being understood solely as individual independence. I | **Pages:** 10-11  **Lines:**154-165  **Pages:** 45  **Lines:** 504-509 |
| *The reliance on English-language studies limits the global representativeness of the findings, potentially excluding important regional perspectives. Additionally, the analysis tends to generalise LMIC contexts, overlooking important variations across countries and healthcare systems.* | We thank you for this important observation. We acknowledge that restricting the review to English-language publications may have resulted in the exclusion of relevant studies published in other languages, particularly from regions where English is not the primary language of scientific communication. This limitation has now been more explicitly acknowledged within the **Limitations** section of the manuscript. We now emphasise that the review identifies broad patterns across LMICs but does not assume homogeneity, and we caution against overgeneralisation of the findings. Furthermore, we recommend that future research should include non-English literature and undertake more context-specific analyses to better understand regional variation in decision-making practices. | **Pages:**47-48  **Lines:** 562-571 |
| *Greater emphasis on service-user voices and a clearer conceptualisation of "involvement" would strengthen the study's interpretive depth.* | We do thank the reviewer for this valuable recommendation. We agree that a clearer conceptualisation of involvement provides a stronger theoretical basis for interpreting the findings. Accordingly, we have revised the **Introduction** to define involvement as a continuum ranging from information sharing and consultation through shared decision-making and supported decision-making to user-led decision-making. This conceptualisation is explicitly linked to the principles of the **UNCRPD** and provides a clearer framework for understanding participation in mental healthcare decision-making. | **Pages:**6  **Lines:**72-84 |

**REVIEWER 4**

| **Reviewer's Comment** | **Author Response** | **Location in Revised Manuscript** |
| --- | --- | --- |
| *The included studies draw upon differing operational definitions of mental illness, ranging from specific diagnoses to broader categories such as severe mental illness. Please clarify how this conceptual heterogeneity was addressed during interpretation and synthesis and whether it influenced comparability across studies.* | We thank the reviewer for this thoughtful observation. We recognise that the included studies adopted diverse conceptualisations of mental health conditions, reflecting variations in diagnostic practice, study design, and local understandings of mental illness. We have therefore expanded the **Methods** section to clarify that the review intentionally adopted a broad conceptualisation of mental health conditions because many qualitative studies did not employ formal psychiatric diagnoses, and several described participants using locally recognised explanatory models rather than internationally standardised diagnostic classifications. The objective of the review was to map patterns of decision-making involvement rather than compare experiences across diagnostic categories. We have also acknowledged in the **Limitations** that future research should explore whether involvement in decision-making differs according to diagnosis | **Pages** 11-12 **line** 179-184 |
| *The review brings together studies using different study designs; including cross-sectional, qualitative, and mixed methods. While this is expected in a scoping review, the manuscript could be made stronger if only the authors explained how the findings obtained from different study designs were integrated within a single analytical framework, and how comparability was handled* | We agree that the inclusion of cross-sectional, qualitative and mixed-methods studies requires clarity regarding how findings were brought together. Consistent with the purpose of a scoping review, we did not treat findings from different study designs as directly comparable or combine them statistically. Rather, the different study designs were considered complementary, with each contributing different forms of evidence relevant to the review question. Quantitative studies provided information on characteristics and associations related to decision-making involvement, while qualitative studies provided greater depth regarding participants' experiences, relationships, barriers and facilitators. Mixed-methods studies contributed both dimensions. |  |
| *The manuscript reports adjusted odds ratios and confidence intervals from one study. Given that scoping reviews are intended to map evidence rather than synthesise effect estimates, please explain the rationale for including this level of statistical detail.* | We appreciate the reviewer raising this important methodological issue. We agree that the primary purpose of a scoping review is to map the breadth and characteristics of the available evidence rather than undertake quantitative synthesis. The adjusted odds ratios and confidence intervals were originally included because they illustrated factors independently associated with decision-making involvement within one of the included studies. To improve consistency across the review, we have revised  the **Results** section to clarify that these findings are presented descriptively as part of the evidence mapping process rather than as pooled effect estimates. We have also ensured greater consistency in the reporting of findings across all included studies to avoid placing disproportionate emphasis on any individual study. | **Pages:**35  **Lines:** 294-298  Pages 47-48 lines 562-569 |
| *There is considerable overlap between the Results and Discussion sections.* | We thank the reviewer for highlighting this issue. We have carefully revised the manuscript to improve the distinction between descriptive reporting and interpretation. The **Results** section now focuses exclusively on presenting the findings from the included studies, while the **Discussion** has been revised to concentrate on interpretation, synthesis of the evidence, comparison with existing literature, and implications for policy, practice, and future research. These revisions have substantially reduced repetition, improved readability, and strengthened the overall structure of the manuscript. | **Pages:**44-47  **Lines:**501-546 |
| *Please reconcile the reporting of the screening process between the narrative and the PRISMA flow diagram.* | We sincerely thank the reviewer for identifying this inconsistency. The PRISMA flow diagram and the accompanying narrative have been carefully reviewed and revised to ensure complete consistency throughout the manuscript. We have corrected the reporting of duplicate records removed, clarified the inclusion of additional records identified through supplementary searches, and ensured that the numbers reported at each stage of the screening process correspond precisely between the text and the PRISMA flow diagram. These revisions improve the transparency and accuracy of the reporting process. | **Pages:**13  **Lines:**200-202 |
| *Table 1 specifies that the study population includes persons with mental health conditions and their caregivers. However, several included studies appear to have recruited healthcare professionals only. Please clarify how these studies met the stated population criterion.* | We thank the reviewer for this important observation. We have revised the **Methods** section to clarify the eligibility criteria. Although the primary focus of the review was on persons with mental health conditions and their caregivers, studies involving healthcare professionals were also considered eligible where they provided direct insight into how service-user and caregiver involvement in mental healthcare decision-making was facilitated, negotiated, constrained, or experienced. We considered these studies relevant because decision-making is inherently relational and frequently occurs through interactions among service users, caregivers, and healthcare professionals. In addition, **Table 1** has been amended to reflect this clarification and ensure consistency between the eligibility criteria and the characteristics of the included studies. | **Pages:10-11**  **Lines:**154-157 |
