# Supplementary material for: Involvement of users and caregivers in mental healthcare decision-making within low-and middle-income countries: A scoping review
Source: PLOS Ment Health. 2026 Oct 30;3(10):e0000712. doi: 10.1371/journal.pmen.0000712 (PMC13722329; doi:10.1371/journal.pmen.0000712)
Supplement: Supplementary file 5 [file pmen.0000712.s005.docx]

**Responses to Reviewer #5**

We thank Reviewer #5 for these thoughtful comments. We have revised the manuscript to clarify the conceptual distinctions between shared, supported, and substitute decision-making; to explain how these concepts were operationalised in the scoping review; to strengthen the rationale for examining caregiver involvement; and to ensure that the wording of the review rationale remains neutral and appropriate for a scoping review.

| **Reviewer comment** | **Response** | **Changes in manuscript** |
| --- | --- | --- |
| **1. The Introduction presents these concepts as part of a continuum, but they are conceptually distinct and may operate at different levels. In particular, shared decision-making generally refers to a process between service users and healthcare professionals, whereas supported decision-making is primarily concerned with preserving an individual's legal capacity and providing support to exercise that capacity. Substitute decision-making concerns situations in which decisions are made on behalf of a person. The authors should clarify these distinctions and explain how each concept is operationalised in the context of this scoping review.** | We thank the reviewer for highlighting the need for greater conceptual clarity. We have revised the Introduction to distinguish clearly between **shared decision-making, supported decision-making, and substitute decision-making**, rather than presenting them as points along a continuum. We now define each concept separately and clarify the actors involved in each form of decision-making. We have also explained how these distinctions **were operationalised in the scoping review**, including how decision-making involving service users, healthcare professionals, caregivers, and other actors was identified and classified during the review process. | **Lines 72–98:** Revised the Background/Introduction to provide conceptual distinctions and definitions of the three decision-making approaches and clarify the actors involved. **Lines 230–234:** Added clarification in the Methods describing how the three concepts were operationalised for the purposes of the review. |
| **2. Although caregivers are included in the title and review objective, the Introduction focuses predominantly on the rights, autonomy, and legal capacity of individuals with mental health conditions. The authors should provide a stronger conceptual and empirical justification for examining caregiver involvement in decision-making, particularly in LMICs where family members may have substantial roles in treatment decisions, care-seeking, consent, and crisis management. It would also be useful to clarify whether "caregivers" refers exclusively to informal/family caregivers or also includes professional caregivers.** | We agree with the reviewer and have strengthened the rationale for examining caregiver involvement. We have added a dedicated paragraph explaining why caregivers are analytically important to understanding decision-making in mental healthcare, particularly in LMIC settings where family members may play substantial roles in care-seeking, treatment decisions, support, and responses to periods of crisis. We have also clarified the meaning of **“caregiver”** in the context of this review, distinguishing informal/family caregivers from professional healthcare providers. This clarification helps ensure that caregiver involvement is examined as a distinct dimension of decision-making rather than being conflated with professional healthcare involvement. | **Lines 100–111:** Added a dedicated paragraph explaining the conceptual and contextual importance of caregiver involvement and explicitly defining **“caregiver.”** |
| **3. In lines 86–88, the authors state that the review aims to better understand “the dominance of substitute decision-making over supported decision-making.” This wording seems to imply that substitute decision-making is already established as dominant in the populations and settings under review. Given that this is a scoping review intended to map the existing evidence, the rationale should be phrased more neutrally. The authors could instead state that the review seeks to examine the extent to which supported and substitute decision-making approaches are reported in LMIC mental healthcare.** | **We agree with the reviewer.** We have revised the wording to avoid presuming that substitute decision-making is dominant in the populations and settings covered by the review. The rationale is now framed more neutrally and reflects the purpose of a scoping review: to **examine and map the extent to which supported and substitute decision-making approaches are reported in LMIC mental healthcare**, rather than to assume the relative dominance of either approach before examining the evidence. | **Lines 92–98:** Revised the statement concerning the “dominance of substitute decision-making over supported decision-making” to adopt neutral, evidence-mapping language appropriate to a scoping review. |

We appreciate Reviewer #5's comments, which have helped us improve the conceptual clarity and methodological transparency of the review. In particular, the revisions now more clearly distinguish the three decision-making concepts, explain the role of caregivers in LMIC mental healthcare, define the actors involved, and align the review rationale with the descriptive and exploratory purpose of a scoping review.

Action AMOS

On behalf of the authors
