# Supplementary material for: Involvement of users and caregivers in mental healthcare decision-making within low-and middle-income countries: A scoping review
Source: PLOS Ment Health. 2026 Oct 30;3(10):e0000712. doi: 10.1371/journal.pmen.0000712 (PMC13722329; doi:10.1371/journal.pmen.0000712)
Supplement: Peer review history

# Author comment: Author response to Decision Letter 0

**DOI:** 10.1371/journal.pmen.0000712.r001

14 Apr 2026

Attachment Submitted filename: draft rebutal letter 18th March.docx

# Peer review: Decision Letter 0

**DOI:** 10.1371/journal.pmen.0000712.r002

**Authors:** Kaaren Mathias

8 Jun 2026

PMEN-D-26-00215

Involvement of users and caregivers in mental healthcare decision-making within low-and middle-income countries: a scoping review

PLOS Mental Health

Dear Dr. Amos,

Thank you for submitting your manuscript to PLOS Mental Health. After careful consideration, we feel that it has merit but does not fully meet PLOS Mental Health’s publication criteria as it currently stands. Therefore, we invite you to submit a revised version of the manuscript that addresses the points raised during the review process.

Please submit your revised manuscript by . If you will need more time than this to complete your revisions, please reply to this message or contact the journal office at mentalhealth@plos.org. When you're ready to submit your revision, log on to https://www.editorialmanager.com/pmen/ and select the 'Submissions Needing Revision' folder to locate your manuscript file.

Please include the following items when submitting your revised manuscript:

- A letter that responds to each point raised by the editor and reviewer(s). You should upload this letter as a separate file labeled 'Response to Reviewers'.
- A marked-up copy of your manuscript that highlights changes made to the original version. You should upload this as a separate file labeled 'Revised Manuscript with Track Changes'.
- An unmarked version of your revised paper without tracked changes. You should upload this as a separate file labeled 'Manuscript'.

Guidelines for resubmitting your figure files are available below the reviewer comments at the end of this letter.

As the corresponding author, your ORCID iD is verified in the submission system and will appear in the published article. PLOS supports the use of ORCID, and we encourage all coauthors to register for an ORCID iD and use it as well. Please encourage your coauthors to verify their ORCID iD within the submission system before final acceptance, as unverified ORCID iDs will not appear in the published article. *Only*  the individual author can complete the verification step; PLOS staff *cannot*  verify ORCID iDs on behalf of authors.

We look forward to receiving your revised manuscript.

Kind regards,

Kaaren R Mathias, PhD

Academic Editor

PLOS Mental Health

**Journal Requirements:**

1. Please amend your detailed Financial Disclosure statement. This is published with the article. It must therefore be completed in full sentences and contain the exact wording you wish to be published.

i. Please clarify all sources of financial support for your study. List the grants, grant numbers, and organizations that funded your study, including funding received from your institution. Please note that suppliers of material support, including research materials, should be recognized in the Acknowledgements section rather than in the Financial Disclosure.

ii. State the initials, alongside each funding source, of each author to receive each grant. For example: "This work was supported by the National Institutes of Health (####### to AM; ###### to CJ) and the National Science Foundation (###### to AM)."

iii. State what role the funders took in the study. If the funders had no role in your study, please state: “The funders had no role in study design, data collection and analysis, decision to publish, or preparation of the manuscript.”

iv. If any authors received a salary from any of your funders, please state which authors and which funders.

2. We note that your Data Availability Statement is currently as follows: All relevant data are within the manuscript and its supporting files.

Please confirm at this time whether or not your submission contains all raw data required to replicate the results of your study. Authors must share the “minimal data set” for their submission. PLOS defines the minimal data set to consist of the data required to replicate all study findings reported in the article, as well as related metadata and methods (https://journals.plos.org/plosone/s/data-availability#loc-minimal-data-set-definition).

For example, authors should submit the following data:

- The values behind the means, standard deviations and other measures reported;

- The values used to build graphs;

- The points extracted from images for analysis.

Authors do not need to submit their entire data set if only a portion of the data was used in the reported study.

If your submission does not contain these data, please either upload them as Supporting Information files or deposit them to a stable, public repository and provide us with the relevant URLs, DOIs, or accession numbers. For a list of recommended repositories, please see https://journals.plos.org/plosone/s/recommended-repositories.

If there are ethical or legal restrictions on sharing a de-identified data set, please explain them in detail (e.g., data contain potentially sensitive information, data are owned by a third-party organization, etc.) and who has imposed them (e.g., an ethics committee). Please also provide contact information for a data access committee, ethics committee, or other institutional body to which data requests may be sent. If data are owned by a third party, please indicate how others may request data access.

3. Please upload separate figure files in .tif or .eps format. Also, remove the figures from your manuscript file but keep the legends.

For more information about figure files please see our guidelines:

https://journals.plos.org/globalpublichealth/s/figures

https://journals.plos.org/globalpublichealth/s/figures#loc-file-requirements

If the reviewer comments include a recommendation to cite specific previously published works, please review and evaluate these publications to determine whether they are relevant and should be cited. There is no requirement to cite these works unless the editor has indicated otherwise.

**Additional Editor Comments (if provided):**

Thank you for this paper which addresses an important gap in research linked to caregiver participation in decision-making in LMIC. Please respond to reviewer comments although some are more salient than others. I would be keen to see this paper include greater detail and thought on the implications of your review for practitioners and more broadly for practice - please add this to your discussion section. I am also wondering if your review explored how the included studies document the processes for participation of caregivers in decision making that are in place and working well (if there is evidence of this) - and if so, would like this to be more strongly reflected in both the findings and discussion - in order to make this paper more useful to practitioners.

thanks

[Note: HTML markup is below. Please do not edit.]

Reviewers' comments:

Reviewer's Responses to Questions

**Comments to the Author**

1. Does this manuscript meet PLOS Mental Health’s publication criteria?

Reviewer #1: Partly

Reviewer #2: Yes

Reviewer #3: Partly

Reviewer #4: Yes

**********

2. Has the statistical analysis been performed appropriately and rigorously?

Reviewer #1: Yes

Reviewer #2: Yes

Reviewer #3: N/A

Reviewer #4: I don't know

**********

3. Have the authors made all data underlying the findings in their manuscript fully available (please refer to the Data Availability Statement at the start of the manuscript PDF file)?

The PLOS Data policy

Reviewer #1: Yes

Reviewer #2: Yes

Reviewer #3: Yes

Reviewer #4: No

**********

4. Is the manuscript presented in an intelligible fashion and written in standard English?

Reviewer #1: Yes

Reviewer #2: Yes

Reviewer #3: Yes

Reviewer #4: Yes

**********

**Reviewer #1:**  The abstract should include the following:Purpose of the study, Methodology used, Findings, discussions, Recommendations, Contributions. General background information needs to be provided with a description of the gap in our knowledge that the study was designed to fill. Methodology needs to be more detailed on how analysis was done. There is an excessive repetition of similar ideas throughout the manuscript, the discussion is overly lengthy and some paragraphs merely restate literature without deeper analysis.

**Reviewer #2:**  The manuscript addresses an important topic. The selection of countries included in the analysis would benefit from further clarification, as this would strengthen the framing and contextual understanding of the findings.

The paper highlights key barriers to participation in meaningful ways and contributes useful insights into structural challenges, while also suggesting interesting areas for future research.

I have added a few minor comments related to terminology, particularly the use of language such as “person with mental illness,” as well as a few small statements throughout the manuscript that may benefit from refinement.

**Reviewer #3:**  This scoping review provides a valuable synthesis of evidence on mental healthcare decision-making in LMICs, highlighting the significant influence of family systems, cultural norms, and structural barriers. While the methodology is robust and aligned with established frameworks, the study would benefit from deeper theoretical grounding, particularly in relation to power dynamics and relational autonomy. The reliance on English-language studies limits the global representativeness of the findings, potentially excluding important regional perspectives. Additionally, the analysis tends to generalize LMIC contexts, overlooking important variations across countries and healthcare systems. Greater emphasis on service user voices and a clearer conceptualization of “involvement” would strengthen the study’s interpretive depth. Overall, the paper makes an important contribution but would be enhanced by more critical analysis, broader inclusion, and stronger integration of theory and policy implications.

The paper has strong potential, but requires substantial conceptual, analytical, and methodological refinement to reach publication standards.

**Reviewer #4:**  Thank you for this well written, timely and relevant paper which makes an important contribution mental health research and advocacy in LMICs. Please receive my comments below, and I hope they will be helpful in strengthening an already worthwhile piece of work.

Major comments

1. The considered studies under the scoping review seem to draw on differing operational definitions of mental illness, ranging from specific diagnoses such as schizophrenia, bipolar disorder, and depression; to even broader categories like "severe mental illness”. Could the authors clarify how such conceptual heterogeneity was reconciled at interpretation and/or synthesis of the findings? Could this have had a bearing on the comparability of the findings across the studies?

2. The review brings together studies using different study designs; including cross-sectional, qualitative, and mixed methods. While this is expected in a scoping review, the manuscript could be made stronger if only the authors explained how the findings obtained from different study designs were integrated within a single analytical framework, and how comparability was handled.

3. It is indicated that a multivariable logistic regression with adjusted odds ratios and confidence intervals from a single study (lines 247-251) was used. However, given that scoping reviews are intended to map the breadth of evidence rather than synthesise effect estimates, could the authors explain what this level of statistical detail adds, and why it is presented for one study but not others?

4. There is an overlap between the results (from line 283 onward) and the discussion, with several repeated passages. Consolidating this content more into one section would tighten the manuscript. If the repetition is intentional and serves a clear purpose, the authors may retain it and briefly justify.

5. The reporting of the screening process (of the studies) might be better presented with clarity if reconciled in the narrative and the PRISMA diagram. The text (lines 159-162) indicates that 10,845 records were excluded from the 21,830 titles screened. It would help readers if the authors stated the number of duplicates removed, clarified where the 46 additional records enter the flow, and ensured the narrative stages and numbers correspond exactly to the diagram.

6. Table one specifies that the study population are persons with a mental health condition and their caregivers. However, there are some included studies which appear to have sampled health professionals only. For example, Tarabey (35 psychiatrists), Abekah-Carter et al. (20 mental health professionals), and Kokota and Stewart (10 mental health experts). Can the authors clarify how these studies meet the stated population criterion?

Minor comments

1. The discussion (line 474) refers to 35 studies reviewed, whereas the abstract, PRISMA diagram, and table 2 indicate 36. Please reconcile.

2. Given that table 2 takes up several pages, a repeating header row on each page could improve readability.

3. Please check carefully the in-text citation numbering against the reference list e.g. Gurung appears as both (31) and (40), and the shared decision making percentages in lines 243-244 seem to be attributed to reference (30) rather than to Ismail (28).

4. The 46 additional records are described either as grey literature or other sources; a single consistent term would help ensure consistence.

5. A few minor typos do require correction, including; an unclosed parenthesis in the abstract i.e. Asia (n = 22, the word Qaulitative in Table 2, p. 30, and Malysia on line 310.

I would like to once again thank the authors for thee engaging and valuable manuscript.

**********

what does this mean?). If published, this will include your full peer review and any attached files.

**Do you want your identity to be public for this peer review?**  If you choose “no”, your identity will remain anonymous but your review may still be made public.

For information about this choice, including consent withdrawal, please see our Privacy Policy

Reviewer #1: **Yes:**  Dr. Nkarenbi Juliette Bih

Reviewer #2: **Yes:**  Sasha Hajj Assaf

Reviewer #3: No

Reviewer #4: **Yes:**  Prichard Denzel Kavuma

**********

[NOTE: If reviewer comments were submitted as an attachment file, they will be attached to this email and accessible via the submission site. Please log into your account, locate the manuscript record, and check for the action link "View Attachments". If this link does not appear, there are no attachment files.]

**Figure Resubmissions:**

# Author comment: Author response to Decision Letter 1

**DOI:** 10.1371/journal.pmen.0000712.r003

15 Aug 2026

Attachment Submitted filename: RESPONSES REVIEWERS letter.docx

# Peer review: Decision Letter 1

**DOI:** 10.1371/journal.pmen.0000712.r004

**Authors:** Kaaren Mathias

16 Sep 2026

PMEN-D-26-00215R1

Involvement of users and caregivers in mental healthcare decision-making within low-and middle-income countries: a scoping review

PLOS Mental Health

Dear Dr. Amos,

Thank you for submitting your manuscript to PLOS Mental Health. After careful consideration, we feel that it has merit but does not fully meet PLOS Mental Health’s publication criteria as it currently stands. Therefore, we invite you to submit a revised version of the manuscript that addresses the points raised during the review process.

Please submit your revised manuscript by 30 September 2026. If you will need more time than this to complete your revisions, please reply to this message or contact the journal office at mentalhealth@plos.org. When you're ready to submit your revision, log on to https://www.editorialmanager.com/pmen/ and select the 'Submissions Needing Revision' folder to locate your manuscript file.

Please include the following items when submitting your revised manuscript:

- A letter that responds to each point raised by the editor and reviewer(s). You should upload this letter as a separate file labeled 'Response to Reviewers'.
- A marked-up copy of your manuscript that highlights changes made to the original version. You should upload this as a separate file labeled 'Revised Manuscript with Track Changes'.
- An unmarked version of your revised paper without tracked changes. You should upload this as a separate file labeled 'Manuscript'.

Guidelines for resubmitting your figure files are available below the reviewer comments at the end of this letter.

As the corresponding author, your ORCID iD is verified in the submission system and will appear in the published article. PLOS supports the use of ORCID, and we encourage all coauthors to register for an ORCID iD and use it as well. Please encourage your coauthors to verify their ORCID iD within the submission system before final acceptance, as unverified ORCID iDs will not appear in the published article. *Only*  the individual author can complete the verification step; PLOS staff *cannot*  verify ORCID iDs on behalf of authors.

We look forward to receiving your revised manuscript.

Kind regards,

Kaaren R Mathias, PhD

Academic Editor

PLOS Mental Health

Journal Requirements:

If the reviewer comments include a recommendation to cite specific previously published works, please review and evaluate these publications to determine whether they are relevant and should be cited. There is no requirement to cite these works unless the editor has indicated otherwise.

Please review your reference list to ensure that it is complete and correct. If you have cited papers that have been retracted, please include the rationale for doing so in the manuscript text, or remove these references and replace them with relevant current references. Any changes to the reference list should be mentioned in the rebuttal letter that accompanies your revised manuscript. If you need to cite a retracted article, indicate the article’s retracted status in the References list and also include a citation and full reference for the retraction notice.

Additional Editor Comments (if provided):

Thank you for your thoughtful revisions to this paper. We have now completed a second round of peer review and one reviewer has requested minor revisions focusing on the concepts operating in this review e.g. caregivers and people with mental health conditions. Please respond to the three minor revisions requested by this reviewer and we will then hope to move quickly to an editorial decision.

[Note: HTML markup is below. Please do not edit.]

Reviewers' comments:

Reviewer's Responses to Questions

**Comments to the Author**

Reviewer #4: All comments have been addressed

Reviewer #5: All comments have been addressed

**********

publication criteria?

Reviewer #4: Yes

Reviewer #5: Yes

**********

3. Has the statistical analysis been performed appropriately and rigorously?

Reviewer #4: Yes

Reviewer #5: Yes

**********

4. Have the authors made all data underlying the findings in their manuscript fully available (please refer to the Data Availability Statement at the start of the manuscript PDF file)?

The PLOS Data policy

Reviewer #4: Yes

Reviewer #5: Yes

**********

5. Is the manuscript presented in an intelligible fashion and written in standard English?

Reviewer #4: Yes

Reviewer #5: Yes

**********

**Review Comments to the Author**

Reviewer #4: I would like to applaud the author for addressing all my comments.

Reviewer #5: The Introduction presents these concepts as part of a continuum, but they are conceptually distinct and may operate at different levels. In particular, shared decision-making generally refers to a process between service users and healthcare professionals, whereas supported decision-making is primarily concerned with preserving an individual's legal capacity and providing support to exercise that capacity. Substitute decision-making concerns situations in which decisions are made on behalf of a person. The authors should clarify these distinctions and explain how each concept is operationalised in the context of this scoping review.

Although caregivers are included in the title and review objective, the Introduction focuses predominantly on the rights, autonomy, and legal capacity of individuals with mental health conditions. The authors should provide a stronger conceptual and empirical justification for examining caregiver involvement in decision-making, particularly in LMICs where family members may have substantial roles in treatment decisions, care-seeking, consent, and crisis management. It would also be useful to clarify whether "caregivers" refers exclusively to informal/family caregivers or also includes professional caregivers.

In lines 86–88, the authors state that the review aims to better understand "the dominance of substitute decision-making over supported decision-making." This wording seems to imply that substitute decision-making is already established as dominant in the populations and settings under review. Given that this is a scoping review intended to map the existing evidence, the rationale should be phrased more neutrally. The authors could instead state that the review seeks to examine the extent to which supported and substitute decision-making approaches are reported in LMIC mental healthcare.

**********

what does this mean?). If published, this will include your full peer review and any attached files.

**Do you want your identity to be public for this peer review?**  If you choose “no”, your identity will remain anonymous but your review may still be made public.

For information about this choice, including consent withdrawal, please see our Privacy Policy

Reviewer #4: **Yes:**  Prichard Denzel Kavuma

Reviewer #5: No

**********

[NOTE: If reviewer comments were submitted as an attachment file, they will be attached to this email and accessible via the submission site. Please log into your account, locate the manuscript record, and check for the action link "View Attachments". If this link does not appear, there are no attachment files.]

# Author comment: Author response to Decision Letter 2

**DOI:** 10.1371/journal.pmen.0000712.r005

30 Sep 2026

Attachment Submitted filename: Responses to Reviewers2.docx

# Editor report: Decision Letter 2

**DOI:** 10.1371/journal.pmen.0000712.r006

**Authors:** Kaaren Mathias

7 Oct 2026

Involvement of users and caregivers in mental healthcare decision-making within low-and middle-income countries: a scoping review

PMEN-D-26-00215R2

Dear Mr Amos,

We are pleased to inform you that your manuscript 'Involvement of users and caregivers in mental healthcare decision-making within low-and middle-income countries: a scoping review' has been provisionally accepted for publication in PLOS Mental Health.

Before your manuscript can be formally accepted you will need to complete some formatting changes, which you will receive in a follow up email. A member of our team will be in touch with a set of requests.

Please note that your manuscript will not be scheduled for publication until you have made the required changes, so a swift response is appreciated.

IMPORTANT: The editorial review process is now complete. PLOS will only permit corrections to spelling, formatting or significant scientific errors from this point onwards. Requests for major changes, or any which affect the scientific understanding of your work, will cause delays to the publication date of your manuscript.

If your institution or institutions have a press office, please notify them about your upcoming paper to help maximize its impact. If they'll be preparing press materials, please inform our press team as soon as possible -- no later than 48 hours after receiving the formal acceptance. Your manuscript will remain under strict press embargo until 2 pm Eastern Time on the date of publication. For more information, please contact mentalhealth@plos.org.

Thank you again for supporting Open Access publishing; we are looking forward to publishing your work in PLOS Mental Health.

Best regards,

Kaaren R Mathias, PhD

Academic Editor

PLOS Mental Health

***********************************************************

Thank you for your responses to the reviewers in the most recent review - I am happy that you have addressed the concerns and happy to go ahead with publication. This is an interesting and important paper - thank you for submitting to PLOS Mental Health.

Reviewer Comments (if any, and for reference):
